# Transcriptomic and metabolomic data of goat ovarian and uterine tissues during sexual maturation

**DOI:** 10.1038/s41597-024-03565-w

**Published:** 2024-07-13

**Authors:** Yanyan Wang, Jianmin Wang, Qing Li, Rong Xuan, Yanfei Guo, Peipei He, Qingling Duan, Shanfeng Du, Tianle Chao

**Affiliations:** 1 Shandong Provincial Key Laboratory of Animal Biotechnology and Disease Control and Prevention, College of Animal Science and Veterinary Medicine, Shandong Agricultural University, Tai’an, Shandong China; 2 Key Laboratory of Efficient Utilization of Non-grain Feed Resources (Co-construction by Ministry and Province), Ministry of Agriculture and Rural Affairs, College of Animal Science and Veterinary Medicine, Shandong Agricultural University, Tai’an, Shandong China

**Keywords:** Transcriptomics, Metabolomics

## Abstract

The ovaries and uterus are crucial reproductive organs in mammals, and their coordinated development ensures the normal development of sexual maturity and reproductive capacity. This study aimed to comprehensively capture the different physiological stages of the goat’s sexual maturation by selecting four specific time points. We collected samples of ovarian and uterine tissues from five female Jining Gray goats at each time point: after birth (D1), 2-month-old (M2), 4-month-old (M4), and 6-month-old (M6). By combining transcriptomic sequencing of 40 samples (including rRNA-depleted RNA-seq libraries with 3607.8 million reads and miRNA-seq libraries with 444.0 million reads) and metabolomics analysis, we investigated the transcriptomic mechanisms involved in reproductive regulation in the ovary and uterus during sexual maturation, as well as the changes in metabolites and their functional potential. Additionally, we analyzed blood hormone indices and uterine tissue sections to examine temporal changes. These datasets will provide a valuable reference for the reproductive regulation of the ovary and uterus, as well as the regulation of metabolites during sexual maturation in goats.

## Background & Summary

Jining Gray (JG) Goat is a local breed found in the southwestern region of Shandong Province, China. It is known for its early sexual maturity, year-round estrus, and high reproductive capacity^1^. In comparison to other breeds, JG goats reach puberty as early as 2 months of age, with sexual maturity occurring significantly earlier (around 3–4 months)^2^. Therefore, major reproductive features, namely ovarian function and hormonal modulation, are already evident during the early growth stages of JG goats. Consequently, these goats can be considered an optimal model for ovarian development examination in livestock at sexual maturity. Furthermore, their exceptional reproductive traits offer more opportunities for studying sexual maturity.

Sexual maturity refers to the stage after birth when an animal undergoes the growth period and reaches the point where it is capable of normal reproduction. The hypothalamus secretes gonadotropin-releasing hormone (GnRH), which induces the pituitary gland to release follicle-stimulating hormone (FSH) and luteinizing hormone (LH), which subsequently accelerates the onset of development, maturation, and ovarian egg release and hormone secretion such as estrogen and progesterone (PROG)^3^. As estrogen levels increase, the endometrium undergoes proliferation and differentiation, entering the proliferative phase. Subsequently, PROG levels rise, leading to the endometrium entering the secretory phase^4^. These changes signify the onset of the menstrual cycle and the arrival of sexual maturity. The ovaries and uterus are vital organs in the goat’s reproductive system. Ovaries synthesize and secrete estrogen and progesterone, as well as maintaining fertility through follicular development and ovulation^5^. The uterus is a target organ for the direct action of ovarian hormones and strictly regulates embryo implantation, pregnancy recognition, and the survival and development of embryos. The physiological functions of the uterus greatly impact the reproductive performance of female livestock, including estrus, mating, conception, and embryo development^6^. The synergistic work of the ovary and uterus facilitates a physiological balance and smooth progression of sexual maturation. This, in turn, lays the foundation for the normal development of reproductive capacity in the offspring. Therefore, the synergistic development of the ovaries and uterus is a key process in the sexual maturation and reproductive development of goats.

Genome-wide sequencing is a widely accepted technology used to comprehensively evaluate the simultaneous changes in animals in response to environmental and dietary induced transcriptional alterations. RNA sequencing (RNA-seq) is a powerful technique for identifying differentially expressed genes and novel transcripts in mammalian reproductive tissues. Specifically, this technology has demonstrated its efficacy in pig gonad^7^, bovine granulosa cell^8^; goat ovary^9^; sheep ovary^10^ and uterus^11^. On the other hand, metabolomics non-specifically identifies and quantitates all low-molecular-weight metabolic end-products (metabolites). It can enhance our comprehension of the downstream metabolic alterations instigated by post-transcriptional regulation, thereby pinpointing the final stage in a series of modifications triggered by external stimuli^12^. Recent studies have shown that oocyte and gonadal development is strictly modulated by an intricate network of metabolic^13–15^. Consequently, the integration of whole-genome sequencing technology and metabolomics offers a robust method for studying the intricate and complex interactions between transcriptional regulation and metabolic processes. This integrated approach holds promise for uncovering the molecular mechanisms that influence reproductive development and exploring metabolic pathways associated with biosynthesis. However, there is currently limited research on the coordinated development of goat ovarian and uterine tissues using both whole-transcriptome and metabolomic studies, especially during the specific stage of sexual maturity. Therefore, in this experiment, we collected ovarian and uterine samples from all the animals on specific days: after birth (D1), as well as 2 (M2), 4 (M4), and 6 months (M6) post birth. These sampling points were chosen to represent distinct physiological phases of goat’s sexual maturation. Through a comprehensive analysis of hormone indices, tissue sections, metabolomic, and transcriptomic data, our goal was to investigate the reproductive regulation of the ovaries and the uterus, and understand the metabolic regulation mechanisms during the sexual maturation process of JG goats.

We presented data from transcriptome sequencing and metabolomics assessment of JG goats ovarian and uterine tissues during their sexual maturation phase. A total of 40 samples were used, resulting in sequencing data of 3607.8 million reads for rRNA-depleted RNA-seq libraries and 444.0 million reads for miRNA-seq libraries. The data provided allow an evaluation of varying developmental stages, from birth to post-sexual maturity, to explore the changes in gene transcription activity and metabolism over time in the ovarian and uterine tissues of goats. Both raw and processed data are freely available and potentially contributing to the understanding of the dynamic molecular regulation processes during sexual maturation in JG goats.

## Methods

### Ethical statement

The research protocol received ethical approval from the review board at Shandong Agricultural University (SDAUA-2023–157).

### Experimental animal and tissue sample collection

The experiment was conducted at the Shandong Jiaxiang JG Goat Breeding Farm (Jining, China). A total of 20 healthy female JG goats were chosen for this study (Table 1), and they were separated into the following 4 age cohorts: D1 (2.60 ± 1.52 days), M2 (2.07 ± 0.04 months), M4 (4.05 ± 0.05 months), and M6 (6.06 ± 0.06 months). Individual age cohort had total of five goats. Food was freely available to all goats, with breeding and management followed the same protocol. Each goat was uniformly slaughtered on its specific cut-off date. Under sterile conditions, the ovarian (O) and uterine (U) tissues from each individual were collected via surgical instruments and rinsed with cold phosphate-buffered saline (PBS). Forty tissue samples were promptly frozen in liquid nitrogen prior to storage at −80 °C until RNA extraction for NGS library construction and metabolite extraction. Additionally, we collected uterine tissues from each age group, preserving them in a 4% formaldehyde fixation solution with the temperature consistently maintained at 4 °C until ready for histological analysis. Where possible, an initial estimate of developmental stage was obtained through dissection and macroscopic examination of the uterus.

**Table 1:** Sample information in this study.

ID	Age	Body Weight (kg)	Ovary Weight (g)	Ovary Length (cm)	Ovary Width (mm)	Ovary Thickness (mm)	Uterine Weight (g)	Cornua Uteri Length (cm)	Uterine Length (cm)	Cervix Length (cm)
**D1-1**	1d	1.84	0.07	6.86	3.94	2.33	1.24	9.24	8.87	16.86
**D1-2**	1d	2.16	0.10	6.56	3.55	2.86	0.89	7.09	9.12	15.42
**D1-3**	3d	1.82	0.06	6.39	3.71	2.75	1.20	9.34	9.64	22.25
**D1-4**	4d	2.18	0.43	10.83	7.13	4.84	1.58	11.02	10.88	22.86
**D1-5**	4d	2.42	0.30	9.59	5.16	4.64	1.11	11.31	10.13	13.82
**M2-1**	2 m	3.78	0.49	9.34	6.06	5.50	1.34	14.66	12.67	20.55
**M2-2**	2 m 2d	4.82	1.12	14.15	8.53	7.82	2.06	14.07	13.13	19.02
**M2-3**	2 m 2d	4.86	0.72	13.17	8.01	7.58	1.72	15.91	18.86	17.19
**M2-4**	2 m 3d	3.86	0.47	12.43	6.43	4.87	2.27	20.91	17.76	15.40
**M2-5**	2 m 3d	4.76	0.84	16.03	7.58	4.89	1.33	14.81	11.69	14.38
**M4-1**	4 m	6.00	0.34	8.27	6.64	4.53	1.74	16.47	18.68	14.74
**M4-2**	4 m	7.14	0.68	9.30	6.94	5.08	2.75	14.43	14.46	8.05
**M4-3**	4 m 2d	7.82	0.41	9.69	6.58	4.44	2.69	17.19	16.37	15.79
**M4-4**	4 m 2d	8.96	0.59	9.95	7.30	5.20	4.08	18.98	17.93	11.31
**M4-5**	4 m 3d	8.20	0.80	9.29	7.24	6.13	9.81	25.73	26.31	13.45
**M6-1**	6 m	9.52	1.24	16.99	7.31	7.66	17.17	32.79	34.38	24.29
**M6-2**	6 m	10.52	1.33	13.32	12.98	6.96	16.61	32.63	33.01	32.70
**M6-3**	6 m 2d	8.44	0.97	14.20	8.59	5.76	9.03	27.33	22.22	15.75
**M6-4**	6 m 3d	8.00	0.70	10.16	7.95	5.99	3.34	17.57	19.14	20.00
**M6-5**	6 m 4d	7.64	0.78	15.03	8.51	6.67	3.30	18.94	21.23	14.40

### Blood sample acquisition and sex hormone content determination

Jugular vein blood (10 mL) was extracted from the experimental goat and transferred to a non-anticoagulant tube, which was maintained in a 37 °C water bath for 1 hour, prior to a 10-min centrifugation at 3,000 r/min. The resulting supernatant was allocated into 2 mL RNase-free tubes, and instantly frozen in liquid nitrogen, prior to transport to the laboratory for storage at −80 °C with proper labeling (ID and sample category) for measurement of sex hormone concentrations. To ensure accurate quantification of hormone concentrations, we selected high-quality enzyme-linked immunosorbent assay (ELISA) kits from Qingdao Mdbio Biotech Co., Ltd., headquartered in Qingdao, China. These kits were specifically chosen for the accurate measurement of specific goat hormones, namely, GnRH, FSH, LH, estradiol (E2), PROG, oxytocin (OT), prolactin (PRL), and relaxin (RLN), following the specific protocols provided with each kit. All kits employ the sandwich ELISA method to quantify hormone levels^16,17^, calculating hormone concentrations from the optical density (OD values) obtained at 450 nm wavelength through a standard curve. Each sample was measured thrice, and comparison assessment was conducted using the mean value along with its standard deviation. Samples underwent a 5-fold dilution to ensure the measurements fell within the linear range of the standard curve, with all samples exhibiting a linear regression correlation coefficient (R-value) exceeding 0.95, thus ensuring precision and reliability of our obtained data. The results of each hormone detection and the standard curves are detailed in Table 2 and Supplementary Fig. 1.

**Table 2:** Serum hormones results at different developmental stages.

Sample	PROG (pg/mL)	LH (mIU/mL)	E2 (pg/mL)	GnRH (pg/mL)	FSH (mIU/mL)	OT (pg/mL)	RLN (pg/mL)	PRL (mIU/L)
**D1-1**	444.12 ± 16.86	5.24 ± 0.12	29.90 ± 0.74	53.37 ± 0.83	3.76 ± 0.13	8.35 ± 0.17	186.26 ± 8.51	254.05 ± 4.10
**D1-2**	490.27 ± 24.50	7.09 ± 0.16	28.17 ± 0.48	56.48 ± 3.66	4.26 ± 0.26	6.17 ± 0.55	145.31 ± 2.61	269.43 ± 10.09
**D1-3**	332.20 ± 10.03	5.57 ± 0.28	28.52 ± 0.70	44.31 ± 2.05	5.25 ± 0.16	8.33 ± 0.54	142.71 ± 1.84	279.79 ± 1.73
**D1-4**	434.62 ± 20.86	7.08 ± 0.31	41.24 ± 1.25	47.16 ± 0.91	5.00 ± 0.31	7.33 ± 0.28	87.74 ± 4.94	310.33 ± 8.36
**D1-5**	362.19 ± 18.56	5.00 ± 0.16	28.73 ± 1.37	43.99 ± 1.73	4.03 ± 0.17	8.40 ± 0.16	118.37 ± 3.00	253.27 ± 3.63
**M2-1**	537.04 ± 16.55	10.51 ± 0.11	43.39 ± 1.87	66.69 ± 2.38	7.80 ± 0.04	10.59 ± 0.29	182.30 ± 11.60	467.14 ± 9.14
**M2-2**	549.97 ± 17.01	9.77 ± 0.36	52.68 ± 1.70	65.63 ± 0.22	6.90 ± 0.28	13.15 ± 1.14	157.48 ± 4.16	454.66 ± 42.24
**M2-3**	481.04 ± 7.53	9.72 ± 0.26	56.31 ± 2.19	62.39 ± 1.05	7.30 ± 0.20	10.41 ± 0.25	135.60 ± 8.22	499.13 ± 22.22
**M2-4**	498.55 ± 20.16	7.92 ± 0.22	38.96 ± 1.14	68.25 ± 1.36	5.32 ± 0.31	16.66 ± 0.08	174.70 ± 6.29	577.92 ± 26.16
**M2-5**	469.92 ± 17.83	9.91 ± 0.53	55.52 ± 1.40	53.72 ± 1.61	5.47 ± 0.16	9.75 ± 0.07	169.72 ± 12.18	418.77 ± 18.60
**M4-1**	523.00 ± 9.29	10.78 ± 0.27	60.37 ± 1.90	77.62 ± 3.77	6.92 ± 0.12	13.30 ± 0.22	121.58 ± 8.90	605.89 ± 26.32
**M4-2**	472.67 ± 14.70	10.67 ± 0.40	58.27 ± 1.90	61.08 ± 0.98	6.60 ± 0.33	14.68 ± 0.46	85.55 ± 12.47	696.05 ± 2.84
**M4-3**	614.30 ± 10.19	10.56 ± 0.30	64.49 ± 3.34	71.82 ± 2.47	6.01 ± 0.27	16.26 ± 0.76	187.70 ± 4.55	565.44 ± 29.95
**M4-4**	581.03 ± 19.13	8.38 ± 0.29	65.82 ± 2.06	83.91 ± 2.60	6.89 ± 0.15	13.91 ± 0.91	190.84 ± 4.16	533.23 ± 38.93
**M4-5**	578.60 ± 2.50	11.60 ± 0.11	47.68 ± 1.87	70.12 ± 2.15	6.74 ± 0.33	15.39 ± 1.94	94.65 ± 3.29	637.10 ± 49.96
**M6-1**	525.88 ± 31.01	8.11 ± 0.50	33.64 ± 1.25	62.95 ± 1.85	5.91 ± 0.23	11.78 ± 0.52	147.20 ± 8.28	330.50 ± 6.73
**M6-2**	476.90 ± 17.93	9.57 ± 0.25	41.20 ± 2.08	57.00 ± 0.24	5.79 ± 0.13	9.04 ± 0.29	109.00 ± 5.61	329.69 ± 1.64
**M6-3**	414.19 ± 11.78	6.85 ± 0.26	54.62 ± 1.70	60.57 ± 0.52	4.87 ± 0.20	11.02 ± 0.43	121.67 ± 9.77	432.51 ± 25.15
**M6-4**	418.19 ± 8.45	9.41 ± 0.41	55.12 ± 1.14	61.77 ± 2.79	5.48 ± 0.23	8.05 ± 0.50	157.41 ± 14.60	358.51 ± 12.55
**M6-5**	424.99 ± 14.80	8.82 ± 0.45	48.89 ± 1.48	50.56 ± 1.87	6.43 ± 0.22	11.59 ± 1.06	95.01 ± 3.83	361.85 ± 27.98

### Histological assessment

**Fig. 1:**
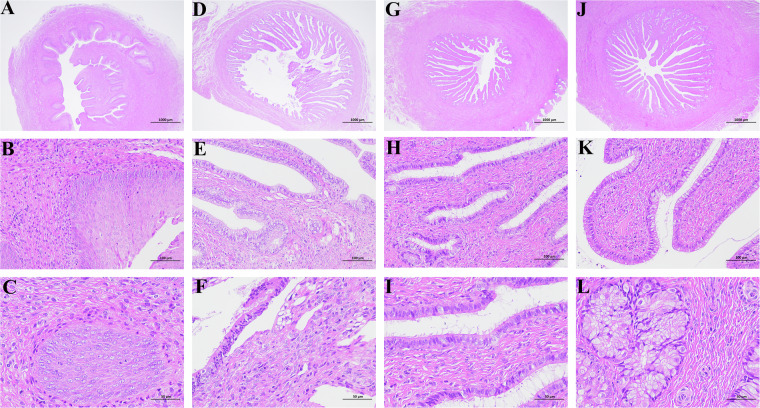
Example images of the uterus of four developmental stages. Bars correspond to 1000 µm (panels **A,B,C** and **D**, 20 × magnification), 100 µm (panels **E,F,G** and **H**, 200× magnification) or 50 µm (panels **I,J,K** and **L**, 400× magnification). Samples shown here are (by goat age): (**A,E** and **I**) after birth (D1); (**B,F** and **J**) 2 months of age (M2); (**C,G** and **K**) 4 months of age (M4); (**D,H** and **L**) 6 months of age (M6).

Fixated uterus tissue underwent dehydration and fixation via rising EtOH (70–100%) and xylene concentrations. individual steps were maintained for a minimum of 30 min. After dehydration, tissues were sequentially placed into three paraffin wax baths at 65 °C for at least 1 hour per bath. Subsequently, the wax-soaked tissue is embedded in the JB-P5 embedding machine. The uterus tissues were sectioned using a RM2016 pathology slicer, with a 4μm slice thickness. Dried sections received Hematoxylin-Eosin staining and observation utilized a Nikon Eclipse E100 light microscope, and images capture employed a NIKON DS-U3 Imaging system. We selected 3 discontinuous sections per sample for observation under a light microscope at 20X, 200X and 400X. The sectional results of uterine tissue from four stages of development, as depicted in Fig. 1., unveil critical indicators of uterine growth: endometrial thickness, glands development, and myometrial thickness^18^. From birth to sexual maturity, we observed notable thickening of the endometrium, enhanced structural integrity, increased gland count, and thickening of the myometrium. These transformations demonstrate the uterus’s continuous maturation and refinement, aiding in our further evaluation of sexual development in JG goats during sexual maturation.

### RNA isolation and quality analysis

Total RNA isolation from 20 uterine tissue and 20 ovarian tissue samples employed TRIzol (Invitrogen, Carlsbad, CA, USA)^19,20^ following the associated directions. TRIzol reagent contains phenol and additives such as 8-hydroxyquinoline, guanidinium thiocyanate, and β-mercaptoethanol, effectively lysing cells and tissues, releasing various types of RNA including non-coding RNA^21^, and inhibiting RNase activity^22^. The experiment was rigorously conducted according to this procedure: (1) For the Trizol-based method, tissue samples of liquid nitrogen homogenate (50–100 mg) were introduced to 1.5 ml Trizol (Invitrogen, California, USA), prior to gentle 5–8 inversions to mix the suspension, and a 5-min maintenance at room temperature (RT) to ensure complete lysis. (2) Add 300 µl chloroform (J.T. Baker, Pennsylvania, USA), then again inverted for 15 secs, prior to a 5-min incubation at RT. Following a 10-min centrifugation at 12,000 × g at 4 °C, the supernatant will divide into three layers: a bottom red phenol-chloroform organic phase, an interphase, and a top colorless aqueous phase, with RNA being predominantly in the aqueous phase. (3) Transfer the aqueous phase to a fresh tube, prior to introducing equal chloroform volume, and repetition of step 2. (4) The top aqueous phase was transferred to a fresh tube containing 500 µl isopropanol (J.T. Baker, Pennsylvania, USA), followed by a 10-min incubation at RT, prior to a 15-min centrifugation at 4 °C, 12,000 × g. Subsequently, the supernatant is discarded. (5) Precipitate is rinsed in 1 ml 75% ethanol (J.T. Baker, Pennsylvania, USA). Centrifuge at 4 °C, 7500 × g for 5 min, and supernatant is removed. (6) Precipitate is air-dried at RT for 5–10 mins, then dissolve the RNA precipitate using an appropriate amount of DEPC-treated water (Qiagen, Hilden, Germany). The RNA concentration, purity and integrity were calculated and checked by a NanoDrop 2000 spectrophotometer (Thermo Scientific, Wilmington, DE, USA) and an Agilent 5400 Bioanalyzer (Agilent, Santa Clara, CA). Currently, the RNA Integrity Number (RIN) is widely used to assess the quality of RNA^23^. RIN values classify RNA samples into 10 predefined integrity categories based on the calculated RIN number for each RNA profile, ranging between 1–10. A RIN = 1 represents completely degraded RNA samples and RIN = 10 represents intact RNA sample. Higher RIN values generally indicate better RNA integrity. RIN values ≥ 8 are optimal for RNA analysis (Table 3)^24^. All RNA sequencing was performed using a single sample.

**Table 3:** The RNA integrity numbers of all samples.

Organ	Sample	Library type	Concentration(ng/ul)	Volume(ul)	Gross(ug)	RNA_RIN
**Ovary**	D1-1	rRNA-depleted RNA-Seq libraries, miRNA-seq libraries	186.00	32.00	5.95	9.10
	D1-2	rRNA-depleted RNA-Seq libraries, miRNA-seq libraries	325.00	32.00	10.40	9.00
	D1-3	rRNA-depleted RNA-Seq libraries, miRNA-seq libraries	313.00	32.00	10.02	9.10
	D1-4	rRNA-depleted RNA-Seq libraries, miRNA-seq libraries	734.00	32.00	23.49	9.40
	D1-5	rRNA-depleted RNA-Seq libraries, miRNA-seq libraries	835.00	32.00	26.72	9.10
	M2-1	rRNA-depleted RNA-Seq libraries, miRNA-seq libraries	697.00	32.00	22.30	9.40
	M2-2	rRNA-depleted RNA-Seq libraries, miRNA-seq libraries	259.00	32.00	8.29	9.10
	M2-3	rRNA-depleted RNA-Seq libraries, miRNA-seq libraries	965.00	32.00	30.88	9.80
	M2-4	rRNA-depleted RNA-Seq libraries, miRNA-seq libraries	413.00	32.00	13.22	9.10
	M2-5	rRNA-depleted RNA-Seq libraries, miRNA-seq libraries	672.00	32.00	21.50	9.30
	M4-1	rRNA-depleted RNA-Seq libraries, miRNA-seq libraries	270.00	32.00	8.64	9.10
	M4-2	rRNA-depleted RNA-Seq libraries, miRNA-seq libraries	119.00	32.00	3.81	8.50
	M4-3	rRNA-depleted RNA-Seq libraries, miRNA-seq libraries	418.00	32.00	13.38	8.90
	M4-4	rRNA-depleted RNA-Seq libraries, miRNA-seq libraries	696.00	32.00	22.27	9.80
	M4-5	rRNA-depleted RNA-Seq libraries, miRNA-seq libraries	569.00	32.00	18.21	9.30
	M6-1	rRNA-depleted RNA-Seq libraries, miRNA-seq libraries	519.00	32.00	16.61	9.50
	M6-2	rRNA-depleted RNA-Seq libraries, miRNA-seq libraries	170.00	32.00	5.44	9.20
	M6-3	rRNA-depleted RNA-Seq libraries, miRNA-seq libraries	418.00	32.00	13.38	9.00
	M6-4	rRNA-depleted RNA-Seq libraries, miRNA-seq libraries	102.00	32.00	3.26	9.00
	M6-5	rRNA-depleted RNA-Seq libraries, miRNA-seq libraries	294.00	32.00	9.41	8.80
**Uterus**	D1-1	rRNA-depleted RNA-Seq libraries, miRNA-seq libraries	1200.00	72.00	86.40	8.10
	D1-2	rRNA-depleted RNA-Seq libraries, miRNA-seq libraries	715.00	32.00	22.88	9.30
	D1-3	rRNA-depleted RNA-Seq libraries, miRNA-seq libraries	747.00	32.00	23.90	9.40
	D1-4	rRNA-depleted RNA-Seq libraries, miRNA-seq libraries	675.00	32.00	21.60	9.20
	D1-5	rRNA-depleted RNA-Seq libraries, miRNA-seq libraries	600.00	32.00	19.20	8.50
	M2-1	rRNA-depleted RNA-Seq libraries, miRNA-seq libraries	420.00	32.00	13.44	8.40
	M2-2	rRNA-depleted RNA-Seq libraries, miRNA-seq libraries	535.00	32.00	17.12	8.70
	M2-3	rRNA-depleted RNA-Seq libraries, miRNA-seq libraries	347.00	32.00	11.10	8.60
	M2-4	rRNA-depleted RNA-Seq libraries, miRNA-seq libraries	239.00	32.00	7.65	8.40
	M2-5	rRNA-depleted RNA-Seq libraries, miRNA-seq libraries	351.00	32.00	11.23	8.70
	M4-1	rRNA-depleted RNA-Seq libraries, miRNA-seq libraries	520.00	32.00	16.64	8.50
	M4-2	rRNA-depleted RNA-Seq libraries, miRNA-seq libraries	343.00	32.00	10.98	9.00
	M4-3	rRNA-depleted RNA-Seq libraries, miRNA-seq libraries	297.00	32.00	9.50	8.70
	M4-4	rRNA-depleted RNA-Seq libraries, miRNA-seq libraries	148.00	32.00	4.74	9.20
	M4-5	rRNA-depleted RNA-Seq libraries, miRNA-seq libraries	92.00	32.00	2.94	8.90
	M6-1	rRNA-depleted RNA-Seq libraries, miRNA-seq libraries	352.00	32.00	11.26	9.60
	M6-2	rRNA-depleted RNA-Seq libraries, miRNA-seq libraries	197.00	32.00	6.30	8.80
	M6-3	rRNA-depleted RNA-Seq libraries, miRNA-seq libraries	396.00	32.00	12.67	9.20
	M6-4	rRNA-depleted RNA-Seq libraries, miRNA-seq libraries	190.00	32.00	6.08	9.00
	M6-5	rRNA-depleted RNA-Seq libraries, miRNA-seq libraries	242.00	32.00	7.74	8.90

### rRNA-depleted RNA-seq and miRNA-seq libraries construction and sequencing

An Epicenter Ribo-Zero™ Removal Kit (Epicenter, Madison, WI, USA) was employed for rRNA elimination, and subsequent rRNA-free residues were purified via ethanol precipitation. Sequences that met our strict quality standards were utilized in library generation and sequencing. The lncRNA and mRNA libraries were created with 3 μg total RNA and a NEBNext® Ultra Directional RNALibrary Prep Kit for Illumina (NEB, USA, Catalog #: E7420S) following kit directions. In a nutshell, we employed probes to eliminate rRNA, thereby purifying mRNA from total RNA. Using divalent cations and high temperature, we fragmented the mRNA in the First Strand Synthesis Reaction Buffer(5X), prior to first strand cDNA synthesis via indiscriminate hexamer primer and M-MuLV Reverse Transcriptase (RNaseH-). Second strand cDNA generation utilized DNA Polymerase I and RNase H, and the resulting cDNA underwent end repair, 3’ end adenylation and adapter ligation. All U-harboring cDNA was eliminated with 3 µL USER Enzyme (NEB, Ipswich, MA, USA), and a 16-cycle PCR enrichment was initiated. Subsequent PCR products underwent purification via AMPure XP beads (Beckman Coulter, Brea, CA, USA), which yielded the final library with strand specificity. After completing library generation, quantification was completed via a Qubit 2.0 Fluorometer (Life Technologies, CA, USA) and diluted libraries to a concentration of 1 ng/ul. Secondly, an Agilent 2100 bioanalyzer (Agilent Technologies, USA) detected the insert size of the library, which was found to be distributed approximately between 250–300 bp. Finally, the qPCR method was used for the precise determination of optimal library concentrations using a Quantification Kit-Illumina NGS Universal (KAPA, # KK4824) on CFX96 Touch Real-Time PCR Detection System (Bio-Rad laboratories, Hercules, CA, USA), ensuring that the effective library concentration was greater than 2 nM. Ultimately, the suitable libraries underwent sequencing on Illumina NovaSeq 6000 platform (Illumina, San Diego, CA, USA) using the PE150 (paired-end 150 bp) strategy.

Forty small RNA libraries were generated via the NEB Next® Multiplex Small RNA Library Prep Set for Illumina® (NEB E7300L) as per the associated directions. First, the 3′ and 5′ adaptors were ligated to the 2 µg of total RNA by T4 RNA ligase for each sample. Subsequently, the first strand cDNA synthesis was performed using M-MuLV Reverse Transcriptase (RNase H-) with the adaptor-ligated RNA as a template. The cDNA was then amplified for 18 cycles using LongAmp Taq 2X Master Mix, SR primer for illumina, and index (X) primer. The resulting PCR products underwent an 8% polyacrylamide gel (100 V, 80 min)-based purification. DNA fragments corresponding to 140~160 bp (the length of small noncoding RNA plus the 3′ and 5′ adaptors) were recovered and dissolved in 8 μL elution buffer for miRNA sequencing library construction.

Once the library was constructed, the Qubit2.0 Fluorometer (Life Technologies, CA, USA) was used for initial quantification. The library was diluted to 1 ng/µl based on the quantitative results. The insert size of the libraries was then detected using an Agilent 2100 Bioanalyzer (Agilent Technologies, USA). The libraries with insert sizes between 18 and 40 bp were accurately quantified using the CFX96 Touch Real-Time PCR Detection System (Bio-Rad Laboratories, Hercules, CA, USA), and the libraries with an effective concentration above 2 nM were used for further sequencing. The qualified libraries underwent sequencing on the Illumina NovaSeq. 6000 platform (Illumina, San Diego, CA, USA), utilizing the SE50 approach (single-end 50 bp, SE50). The aforementioned sequencing was completed by Novogene Co., Ltd. (Beijing, China).

### Sequencing data analysis

The raw imaging data from sequencer was transformed to a sequence file via the CASAVA software (version 1.8.2). This file contained data on both sequence and sequencing quality. To ensure high-quality reads, FastQC software (version 0.11.9) was used to perform quality checks on all samples. For rRNA-depleted RNA-seq libraries, the raw reads filtering utilized the fastp software (version 0.23.1) according to the following parameters: fastp -i in.R1.fq -o out.R1.fq -I in.R2.fq -O out.R2.fq -g -q 5 -u 50 -n 15 -l 150–overlap_diff_limit 1–overlap_diff_percent_limit 10. Next, clean reads were aligned to the reference genome (Capra hircus, ARS1.2) with Hisat2 (version 2.0.5) (parameters:–phred33–rna-strandness RF–dta-cufflinks–un-conc-gz). Transcript were assembled and quantified were utilized using StringTie software (version 1.3.3b). Due to the use of paired-end (PE) sequencing in rRNA-depleted RNA-seq, transcript profiles levels were normalized to FPKM (Fragments Per Kilobase of transcript per Million mapped reads) to facilitate accurate quantification with RSEM (version 1.3.0).

For small RNA libraries, the raw reads were filtered using Cutadapt software (version 1.16) based on the following criteria: (1) exclude low quality (the bases with a sequencing quality (Q) less than 20 account for more than 30% of the entire read); (2) exclude with 10% or more unknown bases; (3) eliminate unique sequences with a length greater than 30 bp or less than 18 bp; (4) filtering out with harboring ploy-N with 5’ adapter contaminants, without 3′ adapter, insert fragments and polyA/T/G/C sequences. Using the Bowtie software (version 1.0.1) filter out repeated sequences and ncRNAs (rRNA, snoRNA, snRNA, tRNA) based on RepeatMasker (https://repeatmasker.org/) and Rfam (https://rfam.org/). Following filtration, the unannotated reads underwent alignment with the goat reference genome by BLAST (version 2.7.1). Finally, the software miREvo (version 1.1) and miRDeep2 (version 2.0.0.7) were integrated to identify miRNAs. Due to the use of single-end (SE) sequencing in miRNA-seq, miRNA expression profiles were normalized to TPM (transcripts per million) with RSEM (version 1.3.0), employing a normalization equation as follows: Normalized expression = (mapped read count / Total reads) * 1,000,000. Principal component analysis (PCA) was conducted on the estimated expression values of the 40 samples using the princomp function (https://www.rdocumentation.org/packages/stats/versions/3.6.2/topics/princomp) in the R package. To assess inter-sample batch effect, we conducted relative log expression (RLE) analysis in RUVSeq (version 1.36.0) package. RLE analysis calculates the read count log-ratio to the median count across samples for individual mRNA, miRNA, or lncRNA.

### Metabolite extraction and LC-MS analysis

We conducted non-specific LC-MS profiling sample analysis. Following the previous reported method^25^, the tissues (100 mg) were initially ground in liquid nitrogen before being resuspended in pre-chilled 80% methanol solvent by well vortex. This solvent effectively disrupts cell membranes, promotes cell lysis to release metabolites, and efficiently extracts broad spectrum metabolites, namely, both polar and non-polar substances^26^, while also demonstrating strong protein precipitation capabilities^27^. Comparative studies have shown that methanol outperforms other solvents in capturing diverse metabolites from complex biological matrices^28^. Subsequently, samples underwent a 5-min maintenance on ice, prior to a 20-min centrifugation at 15,000 × g, 4 °C. A portion of supernatant underwent dilution to 53% methanol with LC-MS grade water, then, samples were placed in a fresh tube for a 20-min centrifugation at 15,000 × g, 4 °C. Lastly, supernatant was used to conduct LC-MS/MS system analysis.

The apparatus and LC-MS setup for this analysis are described below: a Thermo Scientific Q ExactiveTM HF-X mass spectrometer equipped with a dual-sprayer ESI source was connected to a Vanquish UHPLC system from Thermo Fisher, Germany. This Vanquish UHPLC system comprised essential components, encompassing a vacuum degasser, binary pump, thermostated autosampler, and column oven. To comprehensively cover the metabolome, ionization was conducted in both positive and negative ion modes to maximize the identification of two distinct sets of analytes^29^. The setting of instrument parameters was precisely undertaken: a 3.5 kV spray voltage, 320 °C capillary temperature, 35 psi sheath gas flow rate, and 10 L/min auxiliary gas flow rate. The S-lens RF level was accurately maintained at 60, while the temperature of the auxiliary gas heater was precisely set to 350° for optimal performance. Considering the need for effective retention and separation of medium polarity and non-polar metabolites, the Hypersil Gold column (C18) was utilized for sample analysis. The column set at 40 °C (±1 °C), with 0.2 mL/min flow rate, and 17 min run duration. In positive mode, the mobile phase composition for A was 0.1% formic acid in water, while for B it was methanol. In negative ionization mode, mobile phase A involved a solution of 5 mM ammonium acetate particularly with a pH of 9.0, whereas mobile phase B remained methanol. The gradient elution profile was precisely controlled: an initial 2% B for 1.5 minutes, followed by a linear elevation from 2% to 85% B over 3 minutes. This was succeeded by a gradual rise from 85% to 100% B over 10 minutes, and thereafter a decline from 100% to 2% B over 10.1 minutes to restore initial conditions. Finally, a 2% B equilibration step was implemented for 12 minutes to stabilize the system.

### Data processing and metabolite identification

 The UHPLC-MS/MS raw data underwent comprehensive analysis through Compound Discoverer 3.1 developed by Thermo Fisher, which executed peak alignment, peak picking, and quantitation of individual metabolites. Critical parameters encompassed a retention duration tolerance of 0.2 min, actual mass tolerance of 5 ppm, signal intensity tolerance of 30%, signal-to-noise ratio of 3, and a minimum intensity threshold of 100,000. It was attempted to normalize peak intensities to the total spectral intensity, and this normalized value was employed for predicting the molecular formula by amalgamating information from additive ions, molecular ion peaks, and fragment ions. Thereafter, the peaks were meticulously matched against the mzCloud (https://www.mzcloud.org/), mzVault and MassList databases. Compounds exhibiting a coefficient of variation (CV) exceeding 30% in the relative peak area within quality control, abbreviated as QC, samples were accurately excluded, ultimately resulting in the identification and relative quantification of metabolites. The subsequent statistical analyses were undertaken through R 4.3.1 and Python 3.11.4 software.

## Data Records

The raw rRNA-depleted RNA-seq and small RNA-seq read files for ovarian and uterine tissue have been submitted to the NCBI Sequence Read Archive (SRA) under the project numbers PRJNA1091173^30^ and PRJNA1091170^31^, respectively. The raw metabolomics data for ovarian and uterine tissues can be accessed on the MetaboLights database under the accession numbers MTBLS9794^32^ and MTBLS9795^33^, respectively. All provided information can be adopted without restrictions.

## Technical Verification

The RNA sample concentration, purity, and integrity were assessed via an Agilent Bioanalyzer. All samples exhibited good RNA Integrity Number (RIN 9.02 ±  0.38, mean  ±  sd). The quality of the 40 RNA samples is listed in Table 3 and Supplementary Fig. 2. In all, 3607.8 million raw reads were acquired from the rRNA-depleted RNA-seq libraries, and 444.0 million raw reads from the miRNA-seq libraries. Following raw data processing and quality control, a mean of 87,742,600 ± 3,046,275 (mean ± sd) and 10,990,476 ± 815,759 (mean ± sd) clean reads were acquired from the respective libraries. The mapping rates of the rRNA-depleted RNA-seq and miRNA-seq libraries reads against the goat genome were a mean of 94.93% and 96.85%, respectively. The quality of sequencing was evaluated by analyzing the Q30 and GC content distribution in these libraries. Based on our findings, the clean reads quality was adequate for the further analyses. Tables 4 and 5 present the sequencing statistics for samples from two different types of libraries.

**Table 4:** Raw data, clean data, quality and GC content of 40 miRNA-seq libraries.

Organ	Sample_ID	Total reads	Total clean reads	Total mapped reads	Overall alignment rate (%)	“ + ” Mapped sRNA	“−” Mapped sRNA	Q20	Q30	GC (%)
**Ovary**	D1_1	11,795,382	11,634,238	11327672	97.54	5358477 (46.14%)	5969195 (51.40%)	99.37	97.68	49.03
	D1_2	12,583,553	12,391,859	12014107	97.37	4702414 (38.11%)	7311693 (59.26%)	99.26	97.61	49.13
	D1_3	10,730,182	10,389,767	10141035	97.90	1778140 (17.17%)	8362895 (80.73%)	99.03	97.04	49.65
	D1_4	11,345,833	11,238,995	10489538	97.58	4450407 (41.40%)	6039131 (56.18%)	99.32	97.50	49.11
	D1_5	11,927,043	11,822,219	11287211	97.73	4467816 (38.69%)	6819395 (59.05%)	99.41	97.91	49.02
	M2_1	11,561,754	11,438,729	10421607	97.43	6119119 (57.21%)	4302488 (40.23%)	98.82	96.35	50.42
	M2_2	10,964,639	10,862,544	9346596	97.74	6019506 (62.94%)	3327090 (34.79%)	99.36	97.64	51.37
	M2_3	11,667,394	11,544,261	10950915	97.36	6266243 (55.71%)	4684672 (41.65%)	99.34	97.55	50.04
	M2_4	11,229,012	11,146,950	10481098	96.41	6336058 (58.28%)	4145040 (38.13%)	99.22	97.10	50.26
	M2_5	9,988,891	9,899,862	9481457	96.42	5595686 (56.91%)	3885771 (39.52%)	98.94	95.98	50.04
	M4_1	11,123,647	11,014,296	9967354	97.06	6240422 (60.77%)	3726932 (36.29%)	99.08	96.92	50.31
	M4_2	10,192,945	10,110,096	9213977	96.95	5809275 (61.13%)	3404702 (35.82%)	99.15	97.07	50.87
	M4_3	9,897,932	9,569,686	9128991	96.96	5241727 (55.67%)	3887264 (41.29%)	99.11	96.94	49.67
	M4_4	10,594,260	10,538,723	9874737	96.79	5933759 (58.16%)	3940978 (38.63%)	99.15	97.11	50.29
	M4_5	10,345,086	10,285,301	9647667	97.41	5461344 (55.14%)	4186323 (42.27%)	99.14	97.08	50.00
	M6_1	10,078,941	9,966,733	9460125	96.76	5553177 (56.80%)	3906948 (39.96%)	99.09	96.90	50.16
	M6_2	11,733,272	11,636,766	10979906	96.39	5566118 (48.87%)	5413788 (47.53%)	99.11	96.90	50.85
	M6_3	12,497,207	12,404,011	11631634	97.14	7272403 (60.73%)	4359231 (36.40%)	99.13	97.10	50.01
	M6_4	13,227,137	13,127,060	12148588	96.51	5598113 (44.47%)	6550475 (52.04%)	99.23	97.39	50.61
	M6_5	9,963,415	9,910,306	9004866	97.38	5460544 (59.05%)	3544322 (38.33%)	99.16	97.10	50.21
**Uterus**	D1_1	10,395,351	10,325,808	9934650	97.35	3658029 (35.85%)	6276621 (61.51%)	99.01	96.75	49.08
	D1_2	11,703,037	11,628,932	11174290	97.24	4132826 (35.96%)	7041464 (61.27%)	99.17	97.28	49.15
	D1_3	12,174,904	12,123,658	11582938	97.06	4146313 (34.75%)	7436625 (62.32%)	98.79	96.78	49.18
	D1_4	11,083,458	10,991,303	10198466	93.80	4105670 (37.76%)	6092796 (56.04%)	98.30	95.55	49.23
	D1_5	10,305,680	10,234,028	9866827	96.98	3311068 (32.55%)	6555759 (64.44%)	98.52	95.04	49.14
	M2_1	11,875,617	11,689,244	11167499	95.85	4392458 (37.70%)	6775041 (58.15%)	98.69	95.98	48.88
	M2_2	10,196,733	10,144,787	9817393	97.49	5743653 (57.04%)	4073740 (40.45%)	98.35	94.45	49.27
	M2_3	11,474,356	11,370,909	10904649	97.01	6596895 (58.69%)	4307754 (38.32%)	98.71	96.10	49.38
	M2_4	11,091,535	10,988,310	10484930	96.99	6426028 (59.44%)	4058902 (37.55%)	98.84	96.21	49.81
	M2_5	11,318,648	11,217,201	10697132	96.64	6761068 (61.08%)	3936064 (35.56%)	98.60	95.64	49.51
	M4_1	10,028,985	9,983,586	9597191	96.54	5958170 (59.93%)	3639021 (36.61%)	98.62	95.71	49.18
	M4_2	11,274,954	11,221,931	10625021	97.09	6386263 (58.36%)	4238758 (38.73%)	98.72	95.81	49.79
	M4_3	10,836,684	10,704,436	10123545	96.27	6162710 (58.61%)	3960835 (37.67%)	98.57	95.51	49.64
	M4_4	10,074,051	9,971,797	9458742	96.49	4536310 (46.28%)	4922432 (50.21%)	98.66	95.64	50.30
	M4_5	10,454,147	10,360,083	9353732	95.17	5934578 (60.38%)	3419154 (34.79%)	98.67	95.75	51.16
	M6_1	11,430,079	11,309,309	10622284	96.77	5754764 (52.43%)	4867520 (44.35%)	98.84	96.28	49.67
	M6_2	11,316,932	11,198,660	10587118	97.53	6199293 (57.11%)	4387825 (40.42%)	98.82	96.12	49.76
	M6_3	10,349,019	10,204,646	9644454	97.06	5502363 (55.37%)	4142091 (41.69%)	98.38	95.14	49.47
	M6_4	11,462,876	11,365,270	10702278	95.72	5316695 (47.55%)	5385583 (48.17%)	98.68	95.94	50.47
	M6_5	11,706,674	11,652,728	11040722	95.97	5682065 (49.39%)	5358657 (46.58%)	99.10	97.13	50.38

**Table 5:** Raw data, clean data, quality and GC content of 40 rRNA-depleted RNA-Seq libraries.

Organ	Sample_ID	Total reads	Total clean reads	Total mapped reads	Overall alignment rate (%)	Q20	Q30	GC (%)
**Ovary**	D1_1	90,559,960	87,456,638	83,699,952	95.70	96.64	91.09	48.47
	D1_2	88,846,460	85,913,258	82,353,910	95.86	96.77	91.55	48.75
	D1_3	88,391,932	85,715,466	81,853,507	95.49	96.55	91.08	49.12
	D1_4	90,193,710	86,686,748	82,269,124	94.90	96.31	90.75	50.43
	D1_5	88,958,138	86,725,534	83,163,028	95.89	96.58	91.05	49.48
	M2_1	90,757,254	88,688,272	76,006,812	85.70	96.48	90.72	49.20
	M2_2	87,442,138	84,734,622	81,007,796	95.60	96.57	90.98	47.44
	M2_3	90,025,396	87,497,152	84,260,547	96.30	96.56	90.97	49.32
	M2_4	88,029,256	85,384,472	81,947,674	95.97	96.72	91.36	49.10
	M2_5	87,935,742	85,065,058	81,823,789	96.19	96.70	91.25	49.11
	M4_1	91,924,326	89,475,800	85,784,313	95.87	96.60	91.07	48.43
	M4_2	88,530,626	86,111,644	82,163,402	95.41	96.72	91.34	47.58
	M4_3	89,730,126	87,802,624	84,159,947	95.85	96.71	91.29	47.69
	M4_4	86,307,490	83,888,950	80,668,635	96.16	96.39	90.52	47.10
	M4_5	86,004,372	84,327,642	81,106,171	96.18	96.70	91.28	49.05
	M6_1	95,793,254	93,258,238	89,625,383	96.10	96.42	90.64	48.70
	M6_2	89,238,534	86,497,254	82,671,408	95.58	96.57	91.02	48.63
	M6_3	87,059,880	84,824,558	71,156,954	83.89	96.55	90.97	49.09
	M6_4	82,980,246	80,019,902	75,812,839	94.74	96.36	90.62	49.23
	M6_5	87708564	84382672	80477696	95.37	96.19	90.24	49.14
**Uterus**	D1_1	85,450,168	83,022,196	79,534,845	95.80	96.51	90.84	48.99
	D1_2	93,633,838	90,808,110	86,528,912	95.29	96.63	91.27	49.25
	D1_3	87,263,586	85,351,408	81,997,992	96.07	96.72	91.32	48.50
	D1_4	89,380,740	87,092,414	83,485,318	95.86	96.61	91.06	48.88
	D1_5	90,434,262	87,894,724	84,208,884	95.81	96.66	91.29	50.43
	M2_1	87,512,466	85,348,240	81,500,448	95.49	96.78	91.53	47.81
	M2_2	93,946,030	91,020,390	87,278,910	95.89	96.81	91.58	49.35
	M2_3	95,571,878	93,918,972	89,972,440	95.80	96.69	91.32	47.66
	M2_4	93,120,370	90,881,064	86,757,906	95.46	96.62	91.23	47.94
	M2_5	88,794,686	86,508,800	74,379,832	85.98	96.62	91.29	48.38
	M4_1	92,427,514	88,267,104	84,963,488	96.26	96.90	91.70	43.46
	M4_2	93,820,440	91,025,248	86,905,464	95.47	96.90	91.92	50.56
	M4_3	91,692,418	90,070,156	86,357,017	95.88	96.86	91.67	45.38
	M4_4	94,875,686	92,177,554	87,945,510	95.41	96.94	91.84	48.63
	M4_5	92,146,950	89,673,354	85,353,089	95.18	96.98	91.95	49.13
	M6_1	93,580,460	91,888,912	88,345,818	96.14	96.75	91.47	48.45
	M6_2	90,862,438	89,161,514	85,553,201	95.95	96.93	91.89	47.36
	M6_3	92,824,856	90,981,862	87,098,950	95.73	96.75	91.47	48.03
	M6_4	92,900,652	90,968,324	86,973,106	95.61	96.95	91.86	48.76
	M6_5	91,189,112	89,187,168	85,209,946	95.54	96.96	91.96	49.24

**Fig. 2:**
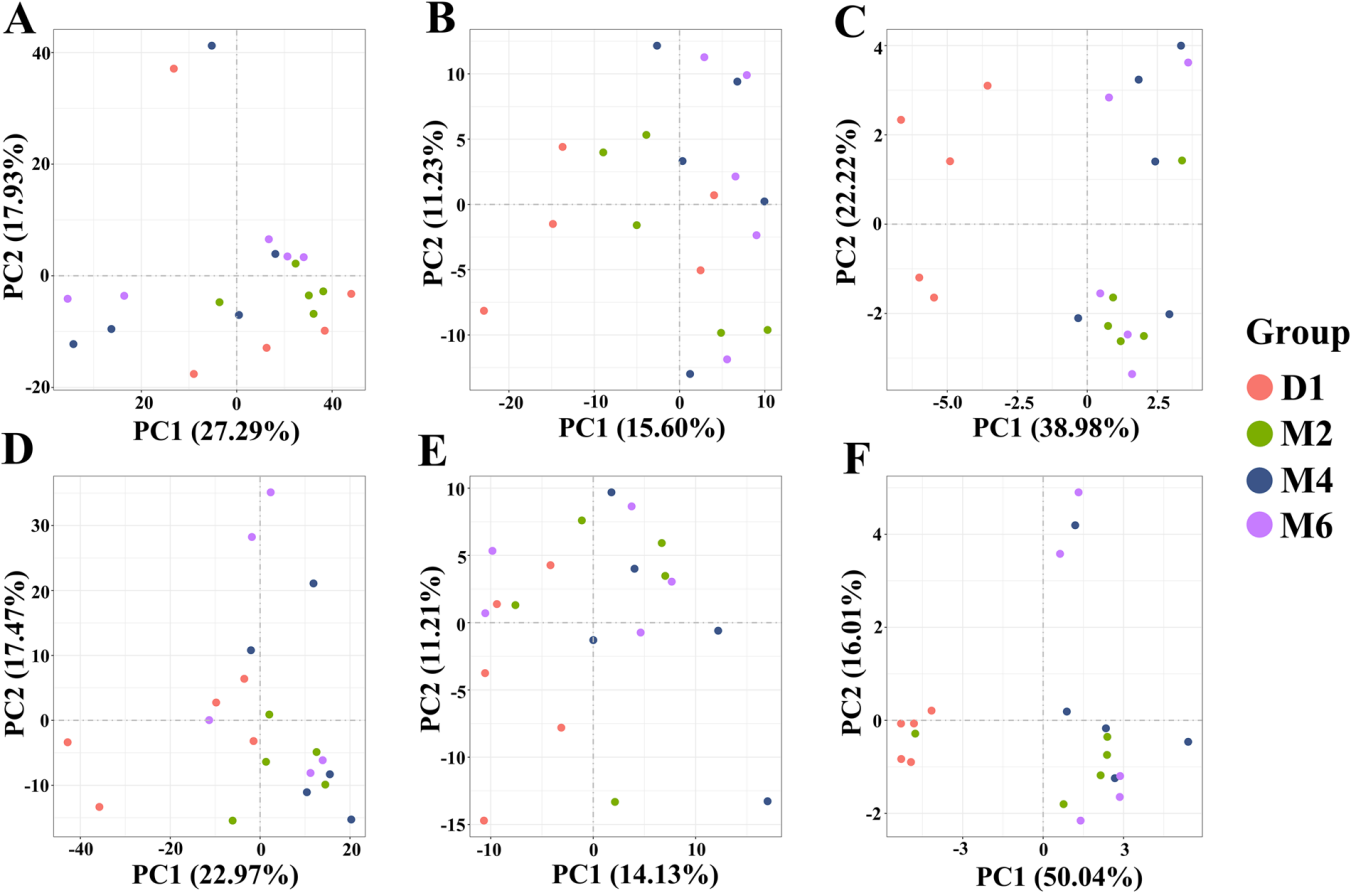
The principal component analysis (PCA) of 40 samples based on estimated expression values. (**A**) The result of the mRNA sequencing of the ovary; (**B**) The result of the lncRNA sequencing of the ovary; (**C**) The result of the miRNA sequencing of the ovary; (**D**) The result of the mRNA sequencing of the uterus; (**E**) The result of the lncRNA sequencing of the uterus; (**F**) The result of the miRNA sequencing of the uterus.

**Fig. 3:**
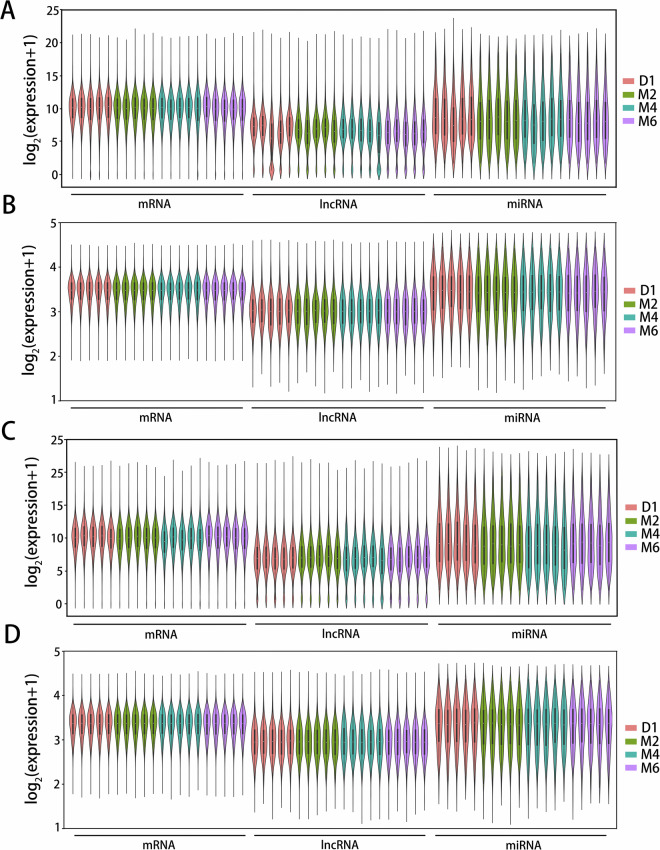
The normalized analysis results. Samples in different time periods were displayed in different colors. (**A**) The violin plots of un-normalized sample RLE for ovarian tissue; (**B**) Violin plots of normalized sample RLE for ovarian tissue; (**C**) Violin plots of un-normalized sample RLE for uterine tissue; (**D**) Violin plots of normalized sample RLE for uterine tissue.

We next conducted PCA, according to the gene expression profile, to reduce the dimensions and retrieve select representative profiles that efficiently represent influences of all genes. Performing PCA on the estimated expression values of the 20 ovarian samples and uterine samples at different developmental stages, respectively, which can allow us to identify the primary variation sources within our data and assess the homogeneity of the populations (Fig. 2). The PCA results showed clustering of QC samples from the same period, indicating the reliability of the test data. Comparable adjustments were performed via relative log expression analysis. As depicted in Fig. 3, violin plots reveal the medium-highly expressed genes distribution, with corresponding median values (circles) and lower to upper quartile range (bars). The first fails to equalize the distributions (Fig. 3A,C), while the second does (Fig. 3B,D). Following RLE quantile adjustment, inter-sample expression profiles become comparable.

**Fig. 4:**
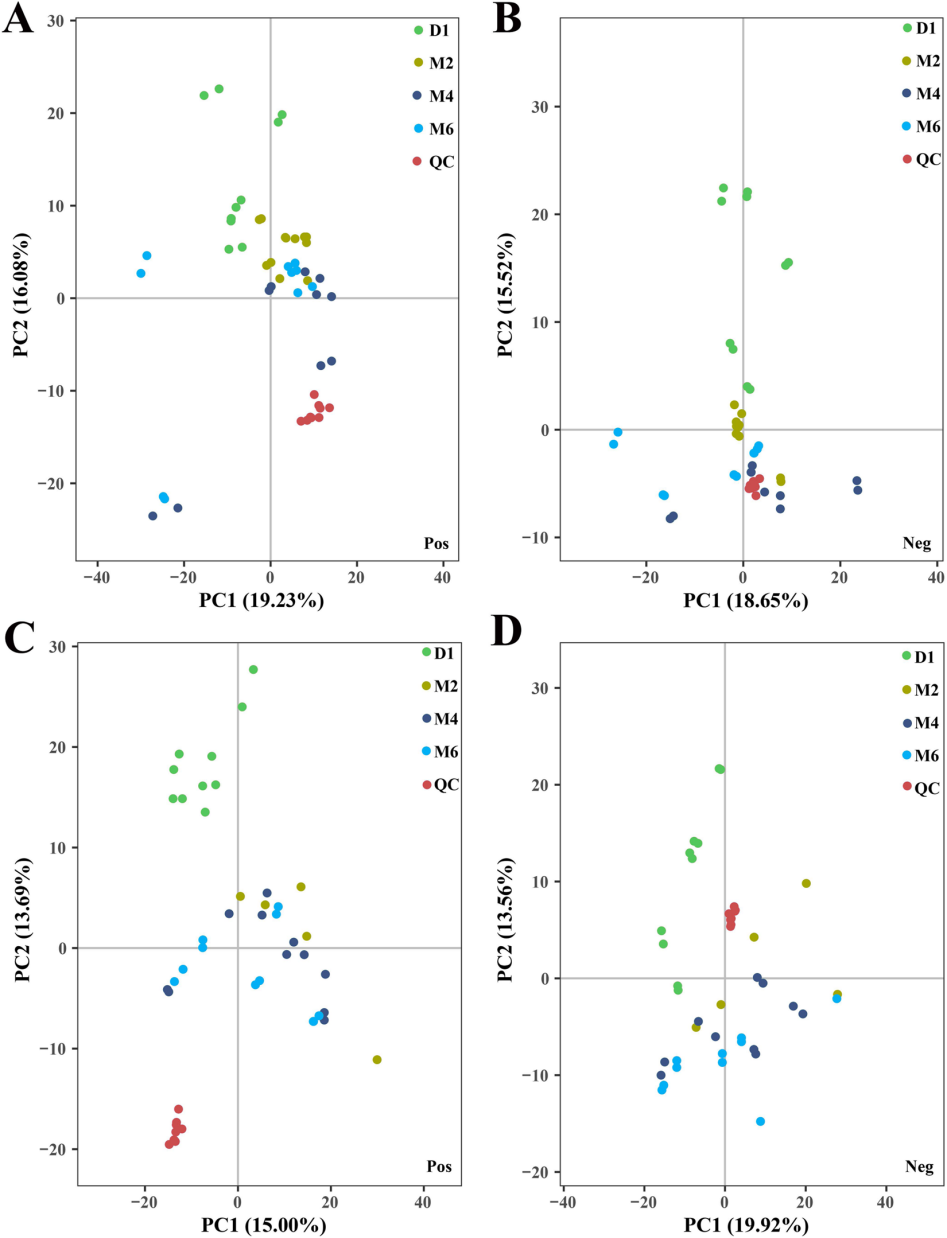
The principal component analysis (PCA) of the ovarian and uterine tissue samples datasets acquired in positive and negative ion mode. (**A**) ovary samples in positive mode; (**B**) ovary samples in negative mode; (**C**) uterus samples in positive mode; (**D**) uterus samples in negative mode.

To guarantee unbiased data generation, we established randomization orders for sample isolation and the sequence of MS runs. All samples from the same batch were analyzed together, with samples randomized according to developmental phases. The total ion chromatograms (TIC) of ovarian and uterine tissue samples captured in both positive (PIM) and negative ion modes (NIM) are displayed in Supplementary Figs. 3,4,5,6. These chromatograms clearly show the degree of separation and sharp peak shapes of different compounds within the chromatography column, indicating good separation efficiency. In ovarian tissues, we identified a total of 1231 metabolites, including 782 in PIM and 449 in NIM. In uterine tissues, altogether 1760 metabolites were identified, with 1136 in PIM and 624 in NIM. These metabolites were categorized into 9 major chemical classes: lipids and lipid-like molecules, organic acids and derivatives, organoheterocyclic compounds, benzenoids and substituted derivatives, nucleosides, nucleotides, and analogues, organic oxygen compounds, phenylpropanoids and polyketides, as well as organic nitrogen compounds and alkaloids and derivatives. Detailed quantitative results, such as the retention times, mass-to-charge ratios, and main classifications of metabolites, are provided in Supplementary Tables 1 and 2. By introducing QC samples, the quality monitoring of mass spectrometry-based metabolomics research is further strengthened^34^. The PCA results of QC samples in both PIM and NIM showed significant clustering, as displayed in Fig. 4, further verifying the reliability and stability of the experimental data.

### Supplementary information

Supplementary Figure

Supplementary Table 1

Supplementary Table 2

## Data Availability

The version and parameter of all bioinformatics tools used in this work are described in the Methods section. During this study, no custom code was used for the curation or validation of the dataset.
