# Supplementary material for: Transcriptomic and metabolomic data of goat ovarian and uterine tissues during sexual maturation
Source: Sci Data. 2024 Jul 13;11:777. doi: 10.1038/s41597-024-03565-w (PMC11246480; doi:10.1038/s41597-024-03565-w)
Supplement: Supplementary file 1 — Supplementary Figure [file 41597_2024_3565_MOESM1_ESM.pdf]

Transcriptomic and metabolomic data of goat ovarian and uterine tissues during sexual maturation

Yanyan Wang[1,2], Jianmin Wang[1,2], Qing Li[1,2], Rong Xuan[1,2], Yanfei Guo[1,2], Peipei He[1,2], Qingling Duan[1,2], Shanfeng Du[1,2], Tianle Chao[1,2]*

1 Shandong Provincial Key Laboratory of Animal Biotechnology and Disease Control and Prevention, College of Animal Science and Veterinary Medicine, Shandong Agricultural University, Tai'an, Shandong, China.
2 Key Laboratory of Efficient Utilization of Non-grain Feed Resources (Co-construction by Ministry and Province), Ministry of Agriculture and Rural Affairs, College of Animal Science and Veterinary Medicine, Shandong Agricultural University, Tai'an, Shandong, China.
corresponding author(s): Tianle Chao (chaotianle@sdau.edu.cn)

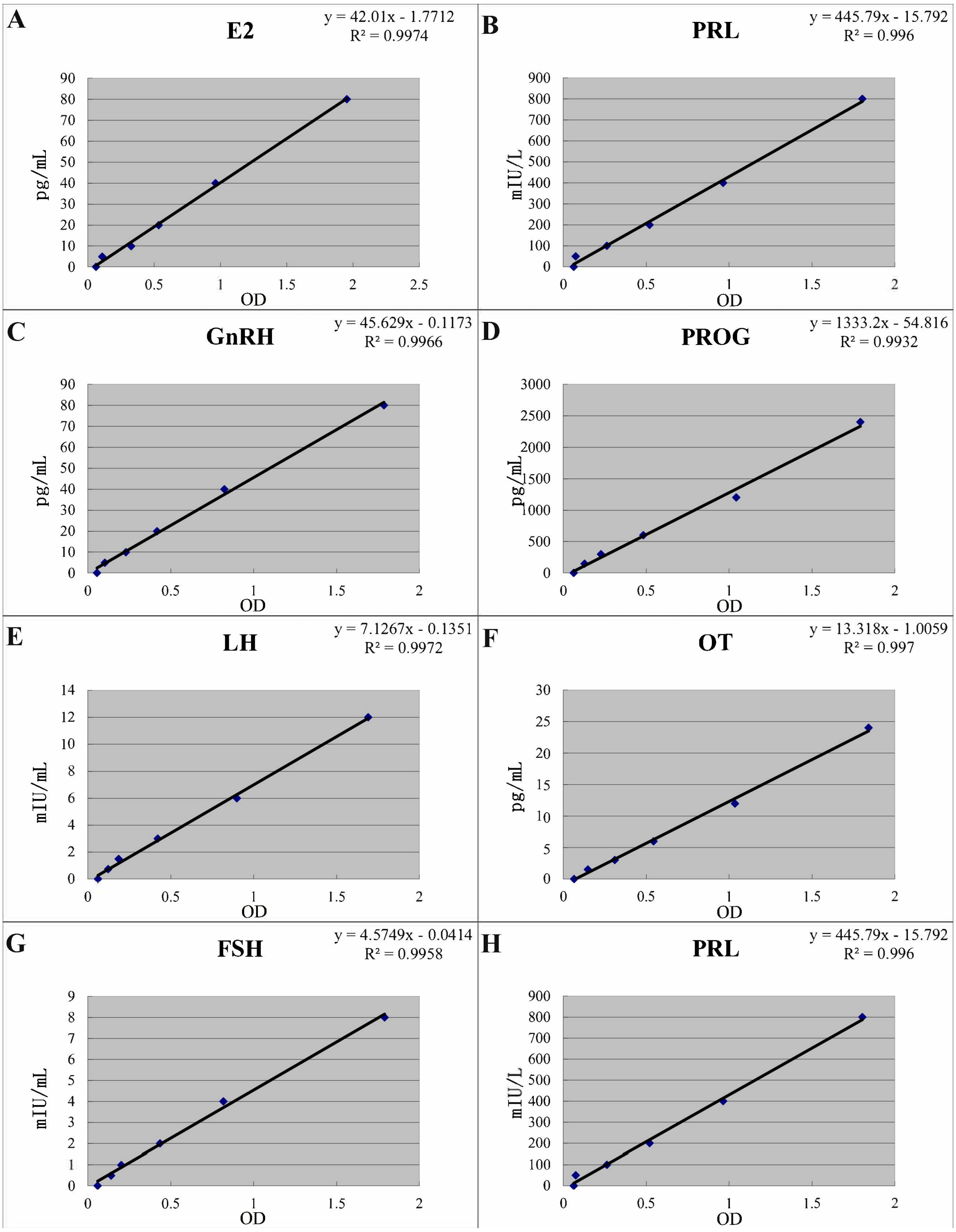

Supplementary Figure 1 The standard curves of various hormones during sexual maturation of Jining Gray goats. (**A**) The standard curve of the estradiol (E2); (**B**) The standard curve of the prolactin (PRL); (**C**) The standard curve of the gonadotropin-releasing hormone (GnRH); (**D**). The standard curve of the progesterone (PROG); (**E**) The standard curve of the luteinizing hormone (LH); (**F**) The standard curve of the oxytocin (OT); (**G**) The standard curve of the follicle-stimulating hormone (FSH); (**H**). The standard curve of the prolactin (PRL).

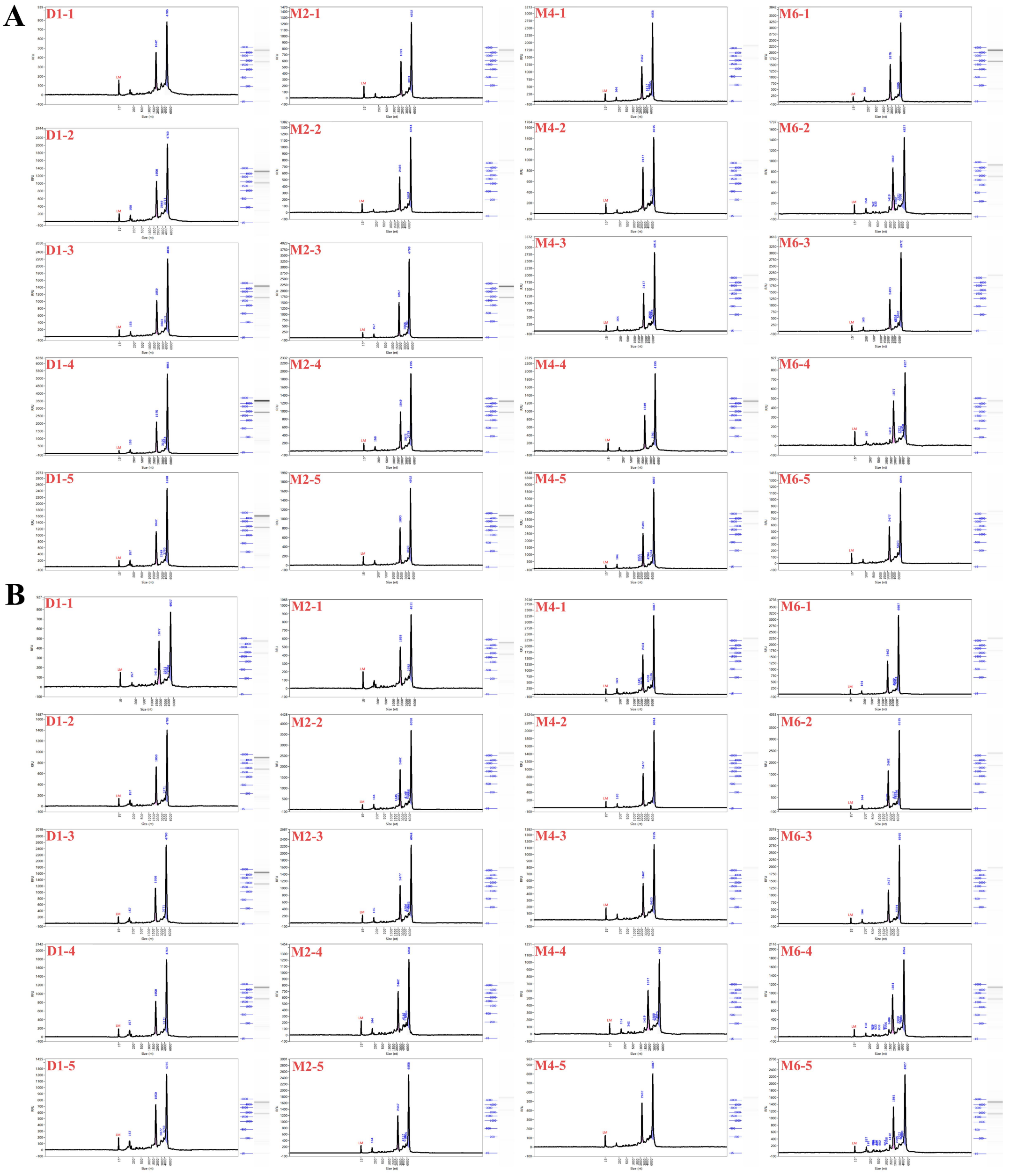

Supplementary Figure 2 RNA quality detection results of ovarian and uterine tissue samples. (A) RNA peak graph of ovarian samples; (B) RNA peak graph of uterine samples.

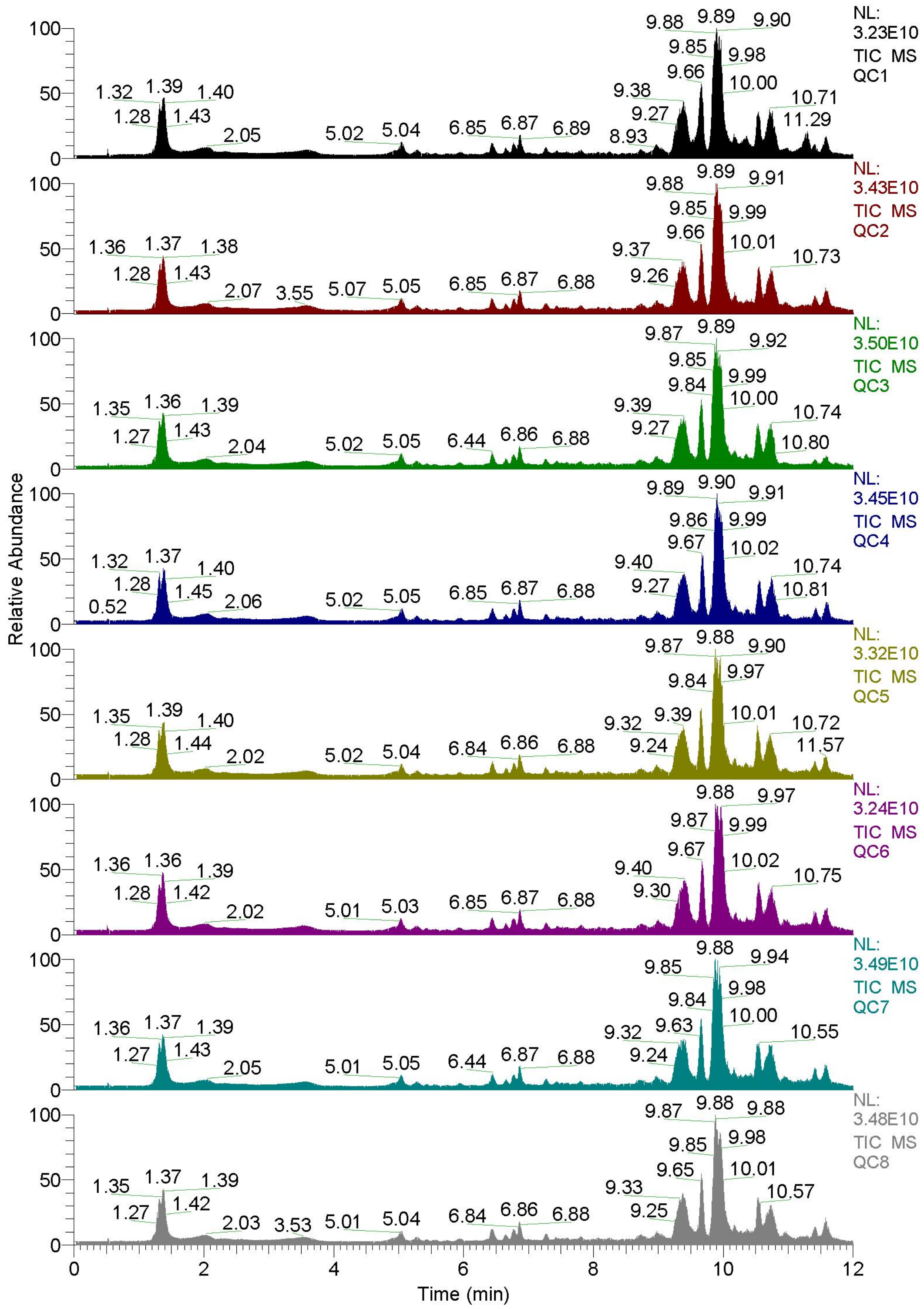
NL: 3.23E10 TIC MS QC1
9.88 9.89 9.90 9.85 9.98 9.66 1.32 1.39 1.40 9.38 10.00 10.71 1.28 1.43 9.27 11.29 2.05 5.02 5.04 6.85 6.87 6.89 8.93
NL: 3.43E10 TIC MS QC2
9.88 9.89 9.91 9.85 9.99 9.66 1.36 1.37 1.38 9.37 10.01 10.73 1.28 1.43 9.26 2.07 3.55 5.07 5.05 6.85 6.87 6.88
NL: 3.50E10 TIC MS QC3
9.87 9.89 9.92 9.85 9.99 9.84 1.35 1.36 1.39 9.39 10.00 10.74 1.27 1.43 9.27 10.80 2.04 5.02 5.05 6.44 6.86 6.88
NL: 3.45E10 TIC MS QC4
9.89 9.90 9.91 9.86 9.99 9.67 1.32 1.37 1.40 9.40 10.02 10.74 1.28 1.45 9.27 10.81 0.52 2.06 5.02 5.05 6.85 6.87 6.88
NL: 3.32E10 TIC MS QC5
9.87 9.88 9.90 9.84 9.97 1.35 1.39 1.40 9.32 9.39 10.01 10.72 1.28 1.44 9.24 11.57 2.02 5.02 5.04 6.84 6.86 6.88
NL: 3.24E10 TIC MS QC6
9.88 9.97 9.87 9.99 9.67 1.36 1.36 1.39 9.40 10.02 10.75 1.28 1.42 9.30 2.02 5.01 5.03 6.85 6.87 6.88
NL: 3.49E10 TIC MS QC7
9.88 9.85 9.94 9.98 9.84 10.00 1.36 1.37 1.39 9.32 9.63 10.55 1.27 1.43 9.24 2.05 5.01 5.05 6.44 6.87 6.88
NL: 3.48E10 TIC MS QC8
9.87 9.88 9.88 9.85 9.98 9.65 10.01 1.35 1.37 1.39 9.33 10.57 1.27 1.42 9.25 2.03 3.53 5.01 5.04 6.84 6.86 6.88
Relative Abundance
0 50 100
0 2 4 6 8 10 12
Time (min)

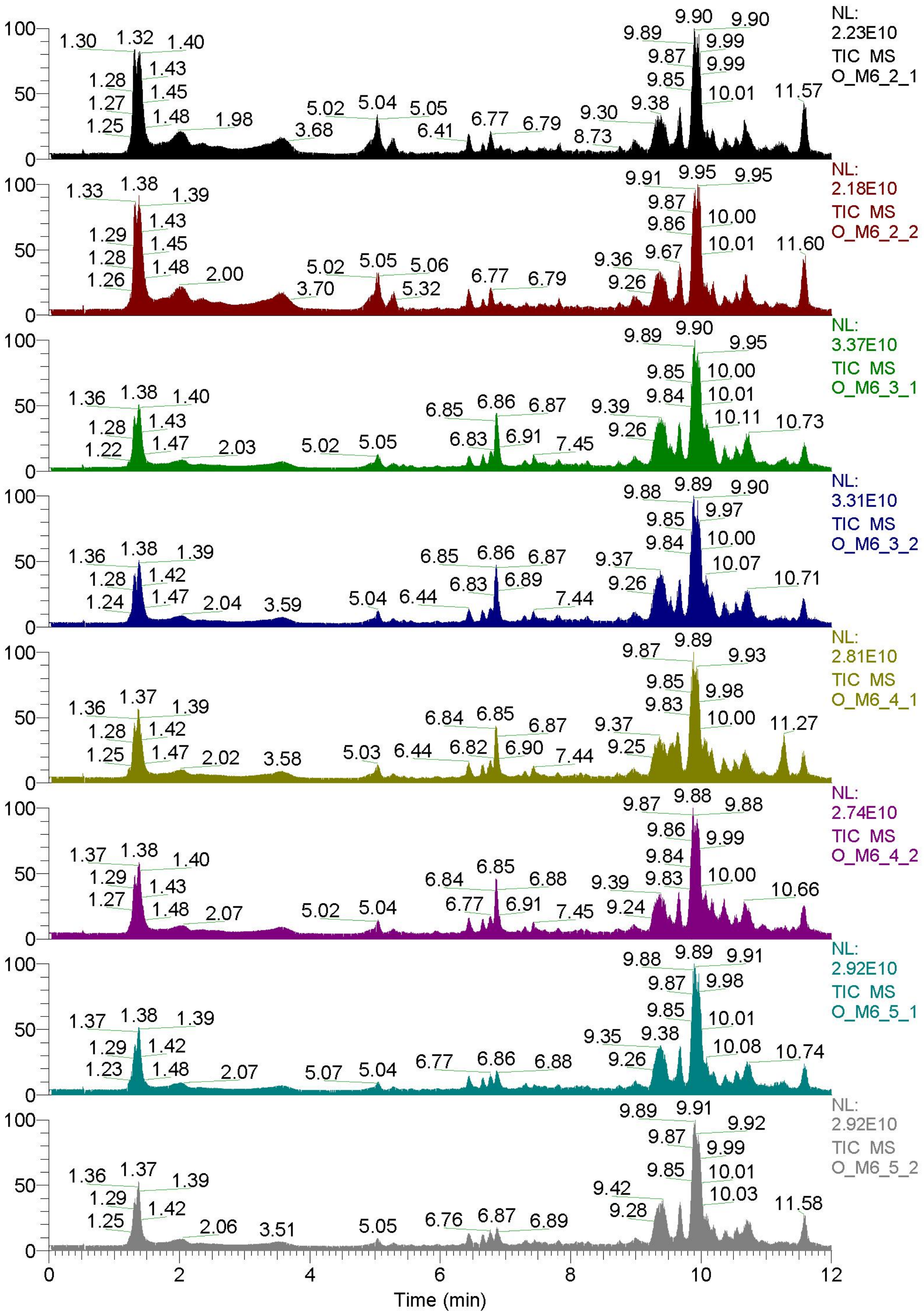

NL:
2.23E10
TIC MS
O_M6_2_1
1.30 1.32 1.40 1.28 1.43 1.27 1.45 1.25 1.48 1.98 3.68 5.02 5.04 5.05 6.41 6.77 6.79 8.73 9.30 9.38 9.89 9.90 9.90 9.99 9.87 9.99 9.85 10.01 11.57
NL:
2.18E10
TIC MS
O_M6_2_2
1.33 1.38 1.39 1.43 1.29 1.45 1.28 1.48 2.00 1.26 3.70 5.02 5.05 5.06 5.32 6.77 6.79 9.36 9.26 9.67 9.91 9.95 9.95 9.87 9.86 10.00 10.01 11.60
NL:
3.37E10
TIC MS
O_M6_3_1
1.36 1.38 1.40 1.28 1.43 1.22 1.47 2.03 5.02 5.05 6.85 6.86 6.87 6.83 6.91 7.45 9.39 9.26 9.89 9.90 9.95 9.85 10.00 9.84 10.01 10.11 10.73
NL:
3.31E10
TIC MS
O_M6_3_2
1.36 1.38 1.39 1.28 1.42 1.24 1.47 2.04 3.59 5.04 6.44 6.85 6.86 6.87 6.83 6.89 7.44 9.37 9.26 9.88 9.89 9.90 9.97 9.85 10.00 9.84 10.07 10.71
NL:
2.81E10
TIC MS
O_M6_4_1
1.36 1.37 1.39 1.28 1.42 1.25 1.47 2.02 3.58 5.03 6.44 6.84 6.85 6.87 6.82 6.90 7.44 9.37 9.25 9.87 9.89 9.93 9.85 9.98 9.83 10.00 11.27
NL:
2.74E10
TIC MS
O_M6_4_2
1.37 1.38 1.40 1.29 1.43 1.27 1.48 2.07 5.02 5.04 6.84 6.85 6.88 6.77 6.91 7.45 9.39 9.24 9.87 9.88 9.88 9.86 9.99 9.84 9.83 10.00 10.66
NL:
2.92E10
TIC MS
O_M6_5_1
1.37 1.38 1.39 1.29 1.42 1.23 1.48 2.07 5.07 5.04 6.77 6.86 6.88 9.35 9.38 9.26 9.88 9.89 9.91 9.87 9.98 9.85 10.01 10.08 10.74
NL:
2.92E10
TIC MS
O_M6_5_2
1.36 1.37 1.39 1.29 1.42 1.25 2.06 3.51 5.05 6.76 6.87 6.89 9.42 9.28 9.89 9.91 9.92 9.87 9.99 9.85 10.01 10.03 11.58
0 50 100
0 2 4 6 8 10 12
Time (min)

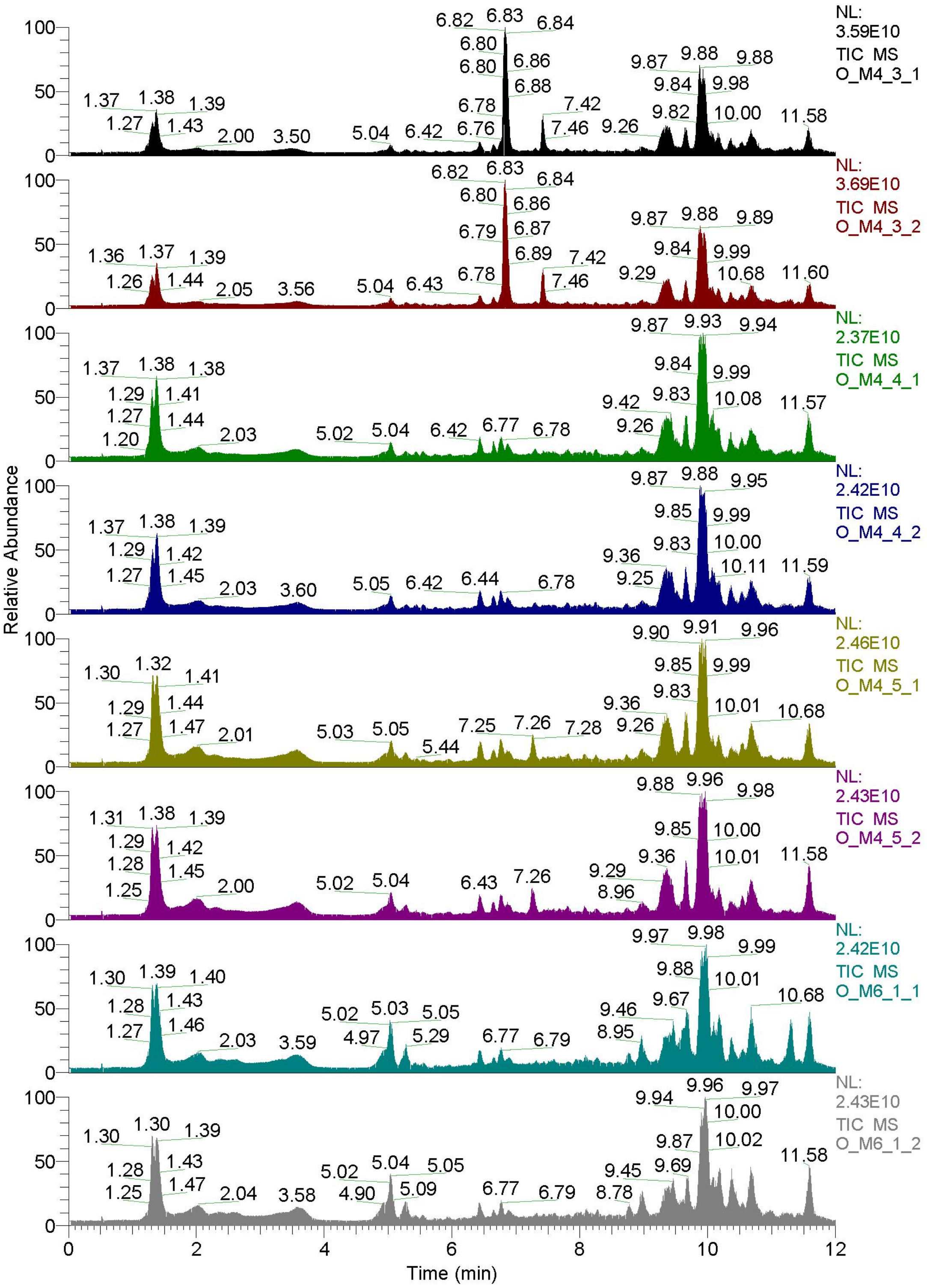
NL: 3.59E10 TIC MS O_M4_3_1
NL: 3.69E10 TIC MS O_M4_3_2
NL: 2.37E10 TIC MS O_M4_4_1
NL: 2.42E10 TIC MS O_M4_4_2
NL: 2.46E10 TIC MS O_M4_5_1
NL: 2.43E10 TIC MS O_M4_5_2
NL: 2.42E10 TIC MS O_M6_1_1
NL: 2.43E10 TIC MS O_M6_1_2
Relative Abundance
Time (min)

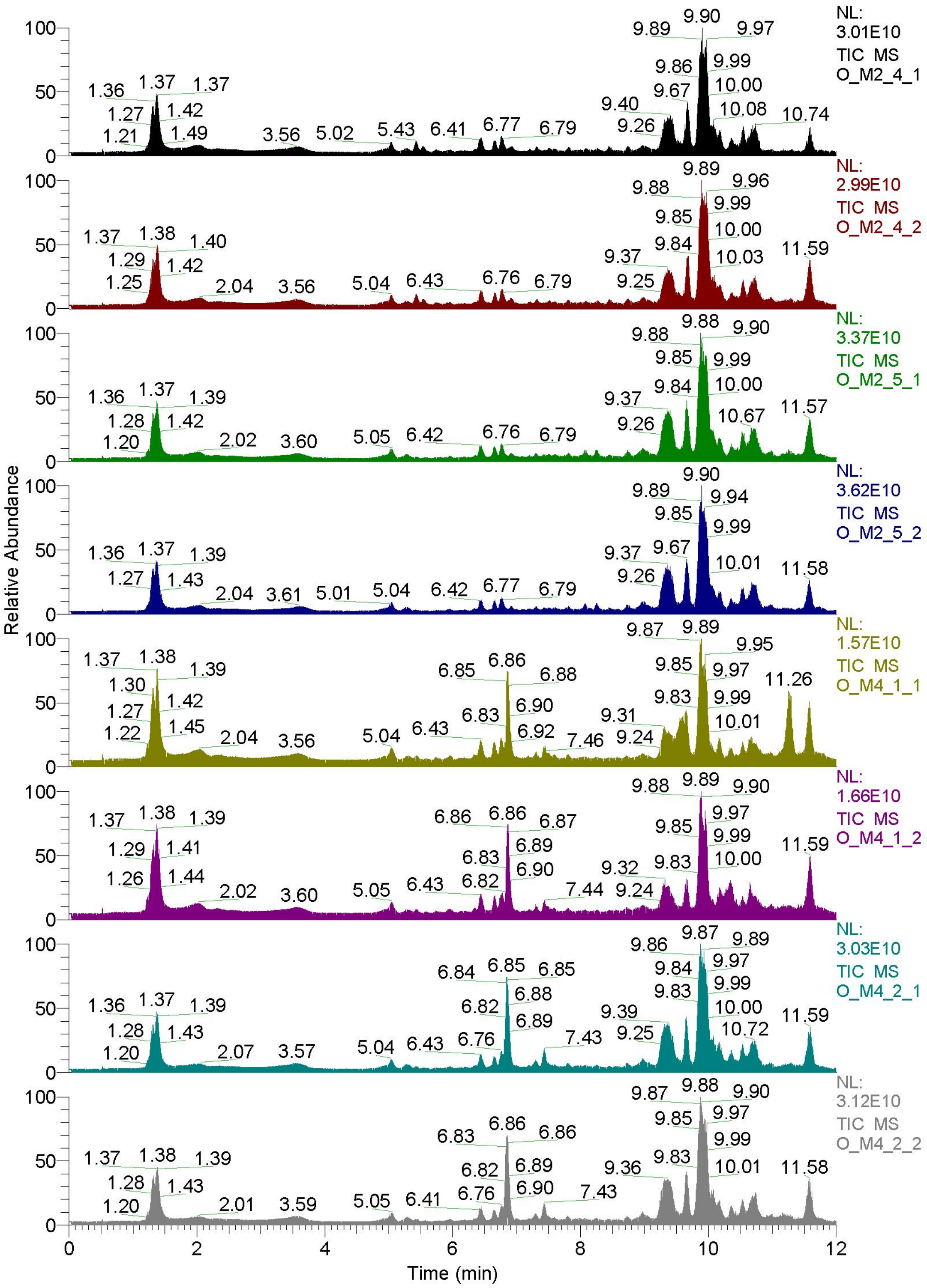

NL: 3.01E10
TIC MS O_M2_4_1
NL: 2.99E10
TIC MS O_M2_4_2
NL: 3.37E10
TIC MS O_M2_5_1
NL: 3.62E10
TIC MS O_M2_5_2
NL: 1.57E10
TIC MS O_M4_1_1
NL: 1.66E10
TIC MS O_M4_1_2
NL: 3.03E10
TIC MS O_M4_2_1
NL: 3.12E10
TIC MS O_M4_2_2
Relative Abundance
Time (min)

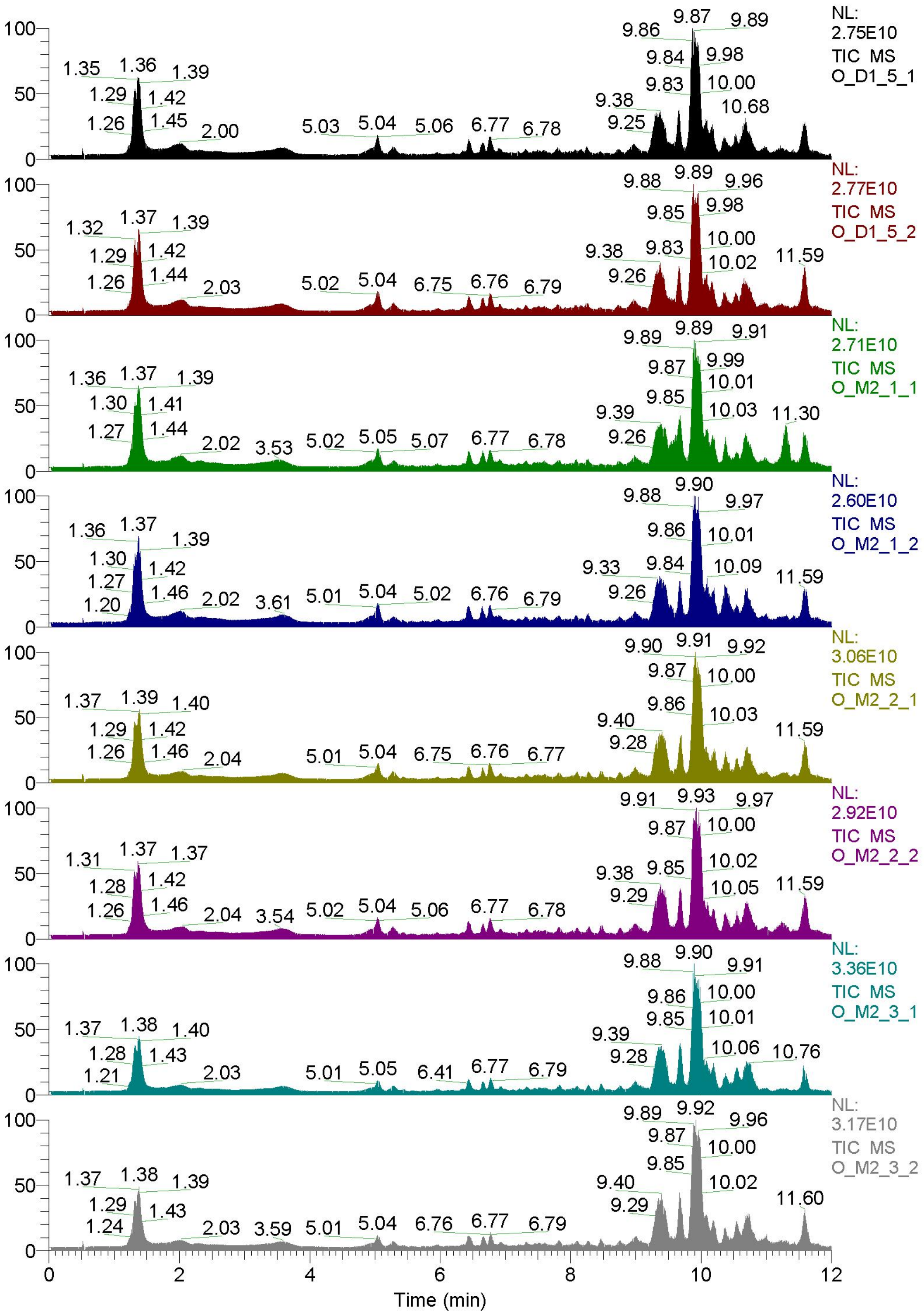

NL: 2.75E10 TIC MS O_D1_5_1
1.35 1.36 1.39 1.29 1.42 1.26 1.45 2.00 5.03 5.04 5.06 6.77 6.78 9.86 9.87 9.89 9.84 9.98 9.83 10.00 9.38 9.25 10.68
NL: 2.77E10 TIC MS O_D1_5_2
1.32 1.37 1.39 1.29 1.42 1.26 1.44 2.03 5.02 5.04 6.75 6.76 6.79 9.88 9.89 9.96 9.85 9.98 9.38 9.83 10.00 10.02 9.26 11.59
NL: 2.71E10 TIC MS O_M2_1_1
1.36 1.37 1.39 1.30 1.41 1.27 1.44 2.02 3.53 5.02 5.05 5.07 6.77 6.78 9.89 9.89 9.91 9.87 9.99 10.01 9.39 9.85 10.03 11.30 9.26
NL: 2.60E10 TIC MS O_M2_1_2
1.36 1.37 1.39 1.30 1.42 1.27 1.46 1.20 2.02 3.61 5.01 5.04 5.02 6.76 6.79 9.88 9.90 9.97 9.86 10.01 9.33 9.84 10.09 11.59 9.26
NL: 3.06E10 TIC MS O_M2_2_1
1.37 1.39 1.40 1.29 1.42 1.26 1.46 2.04 5.01 5.04 6.75 6.76 6.77 9.90 9.91 9.92 9.87 10.00 9.86 10.03 9.40 9.28 11.59
NL: 2.92E10 TIC MS O_M2_2_2
1.31 1.37 1.37 1.28 1.42 1.26 1.46 2.04 3.54 5.02 5.04 5.06 6.77 6.78 9.91 9.93 9.97 9.87 10.00 9.85 10.02 9.38 10.05 9.29 11.59
NL: 3.36E10 TIC MS O_M2_3_1
1.37 1.38 1.40 1.28 1.43 1.21 2.03 5.01 5.05 6.41 6.77 6.79 9.88 9.90 9.91 9.86 10.00 9.85 10.01 9.39 10.06 9.28 10.76
NL: 3.17E10 TIC MS O_M2_3_2
1.37 1.38 1.39 1.29 1.43 1.24 2.03 3.59 5.01 5.04 6.76 6.77 6.79 9.89 9.92 9.96 9.87 10.00 9.85 9.40 10.02 9.29 11.60
100
50
0
0 2 4 6 8 10 12
Time (min)

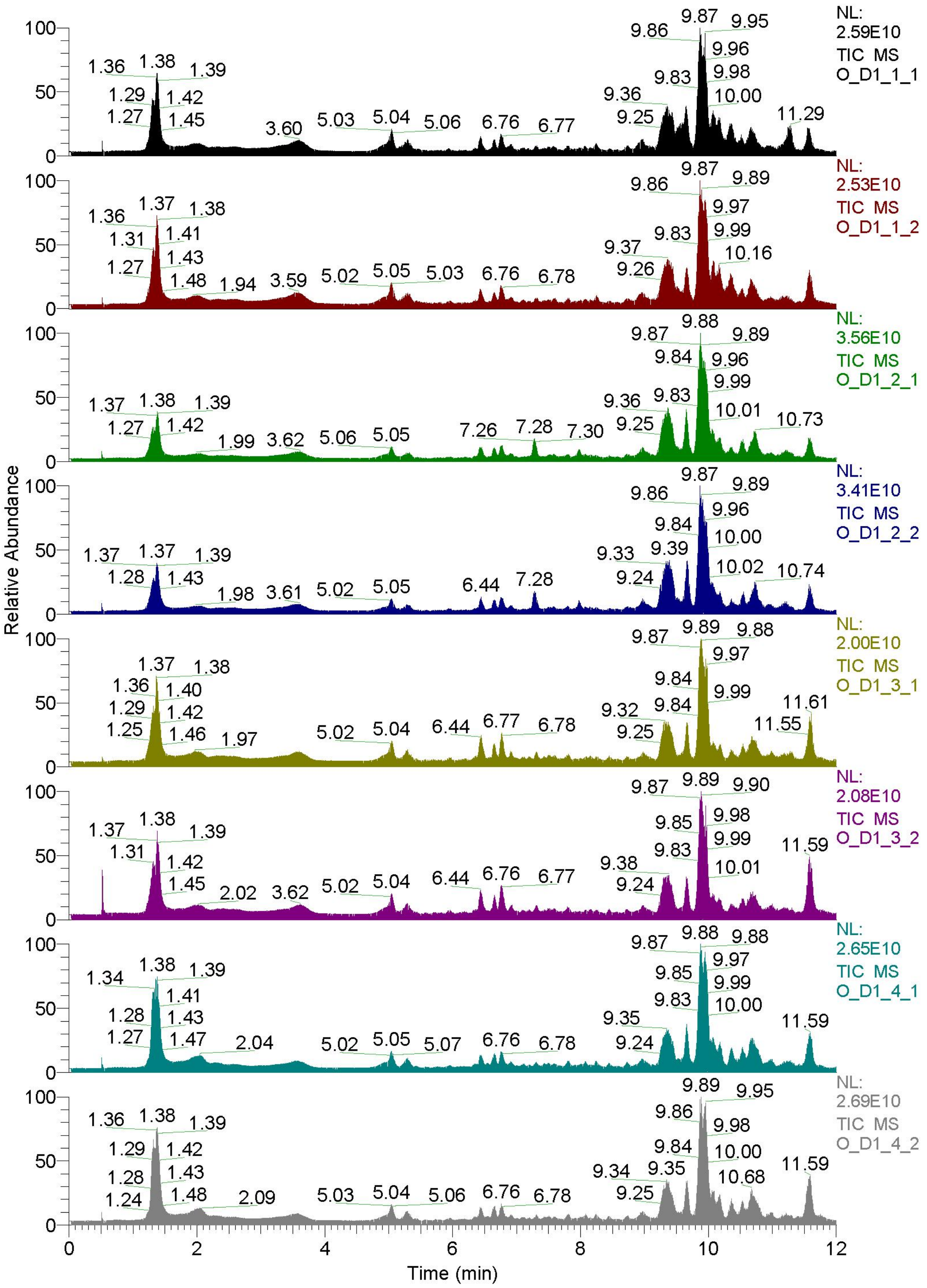
NL: 2.59E10 TIC MS O_D1_1_1
1.36 1.38 1.39 1.29 1.42 1.27 1.45 3.60 5.03 5.04 5.06 6.76 6.77 9.86 9.87 9.95 9.96 9.83 9.98 9.36 10.00 9.25 11.29
NL: 2.53E10 TIC MS O_D1_1_2
1.36 1.37 1.38 1.31 1.41 1.43 1.27 1.48 1.94 3.59 5.02 5.05 5.03 6.76 6.78 9.86 9.87 9.89 9.97 9.83 9.99 9.37 10.16 9.26
NL: 3.56E10 TIC MS O_D1_2_1
1.37 1.38 1.39 1.27 1.42 1.99 3.62 5.06 5.05 7.26 7.28 7.30 9.87 9.88 9.89 9.84 9.96 9.99 9.36 9.83 10.01 9.25 10.73
NL: 3.41E10 TIC MS O_D1_2_2
1.37 1.37 1.39 1.28 1.43 1.98 3.61 5.02 5.05 6.44 7.28 9.86 9.87 9.89 9.96 9.84 10.00 9.33 9.39 10.02 10.74 9.24
NL: 2.00E10 TIC MS O_D1_3_1
1.37 1.38 1.36 1.40 1.29 1.42 1.25 1.46 1.97 5.02 5.04 6.44 6.77 6.78 9.87 9.89 9.88 9.97 9.84 9.99 9.32 9.84 11.61 9.25 11.55
NL: 2.08E10 TIC MS O_D1_3_2
1.37 1.38 1.39 1.31 1.42 1.45 2.02 3.62 5.02 5.04 6.44 6.76 6.77 9.87 9.89 9.90 9.98 9.85 9.99 9.83 11.59 9.38 10.01 9.24
NL: 2.65E10 TIC MS O_D1_4_1
1.34 1.38 1.39 1.41 1.28 1.43 1.27 1.47 2.04 5.02 5.05 5.07 6.76 6.78 9.87 9.88 9.88 9.97 9.85 9.99 9.83 10.00 9.35 11.59 9.24
NL: 2.69E10 TIC MS O_D1_4_2
1.36 1.38 1.39 1.29 1.42 1.28 1.43 1.24 1.48 2.09 5.03 5.04 5.06 6.76 6.78 9.89 9.95 9.86 9.98 9.84 10.00 9.34 9.35 10.68 11.59 9.25
Relative Abundance
0 50 100
Time (min)
0 2 4 6 8 10 12

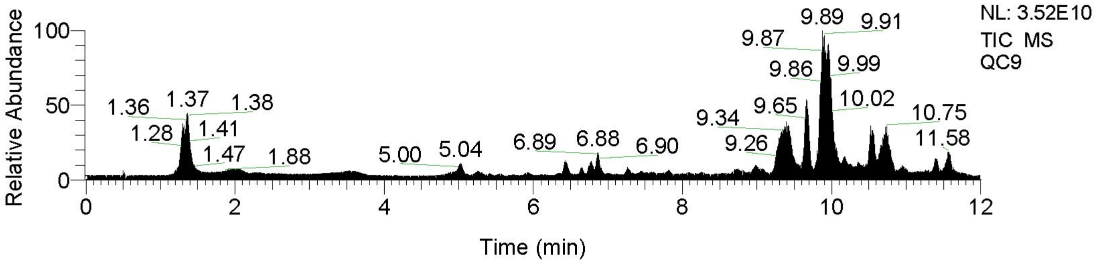

Supplementary Figure 3 The total ion chromatograms (TIC) of each ovarian tissue sample captured in positive ion mode.

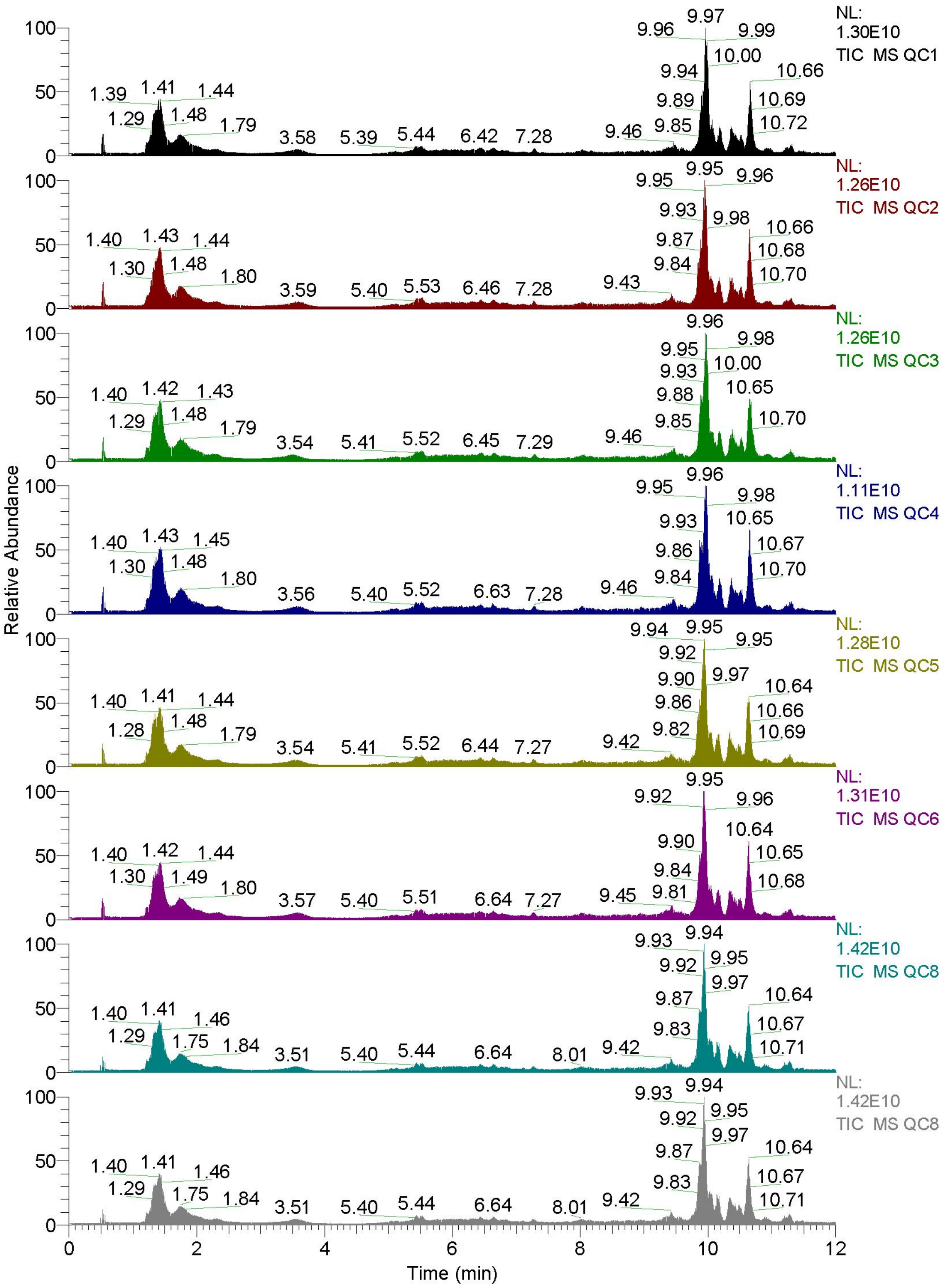

NL: 1.30E10 TIC MS QC1
9.96 9.97 9.99 10.00 9.94 10.66 9.89 10.69 9.85 10.72 9.46 7.28 6.42 5.44 5.39 3.58 1.79 1.48 1.29 1.39 1.41 1.44
NL: 1.26E10 TIC MS QC2
9.95 9.95 9.96 9.93 9.98 10.66 9.87 10.68 9.84 10.70 9.43 7.28 6.46 5.53 5.40 3.59 1.80 1.48 1.30 1.40 1.43 1.44
NL: 1.26E10 TIC MS QC3
9.96 9.95 9.98 9.93 10.00 10.65 9.88 9.85 10.70 9.46 7.29 6.45 5.52 5.41 3.54 1.79 1.48 1.29 1.40 1.42 1.43
NL: 1.11E10 TIC MS QC4
9.95 9.96 9.98 9.93 10.65 9.86 10.67 9.84 10.70 9.46 7.28 6.63 5.52 5.40 3.56 1.80 1.48 1.30 1.40 1.43 1.45
NL: 1.28E10 TIC MS QC5
9.94 9.95 9.95 9.92 9.90 9.97 10.64 9.86 10.66 9.82 10.69 9.42 7.27 6.44 5.52 5.41 3.54 1.79 1.48 1.28 1.40 1.41 1.44
NL: 1.31E10 TIC MS QC6
9.92 9.95 9.96 10.64 9.90 10.65 9.84 9.81 10.68 9.45 7.27 6.64 5.51 5.40 3.57 1.80 1.49 1.30 1.40 1.42 1.44
NL: 1.42E10 TIC MS QC8
9.93 9.94 9.92 9.95 9.97 9.87 10.64 9.83 10.67 10.71 9.42 8.01 6.64 5.44 5.40 3.51 1.84 1.75 1.29 1.40 1.41 1.46
NL: 1.42E10 TIC MS QC8
9.93 9.94 9.92 9.95 9.97 9.87 10.64 9.83 10.67 10.71 9.42 8.01 6.64 5.44 5.40 3.51 1.84 1.75 1.29 1.40 1.41 1.46
Relative Abundance
0 50 100
Time (min)
0 2 4 6 8 10 12

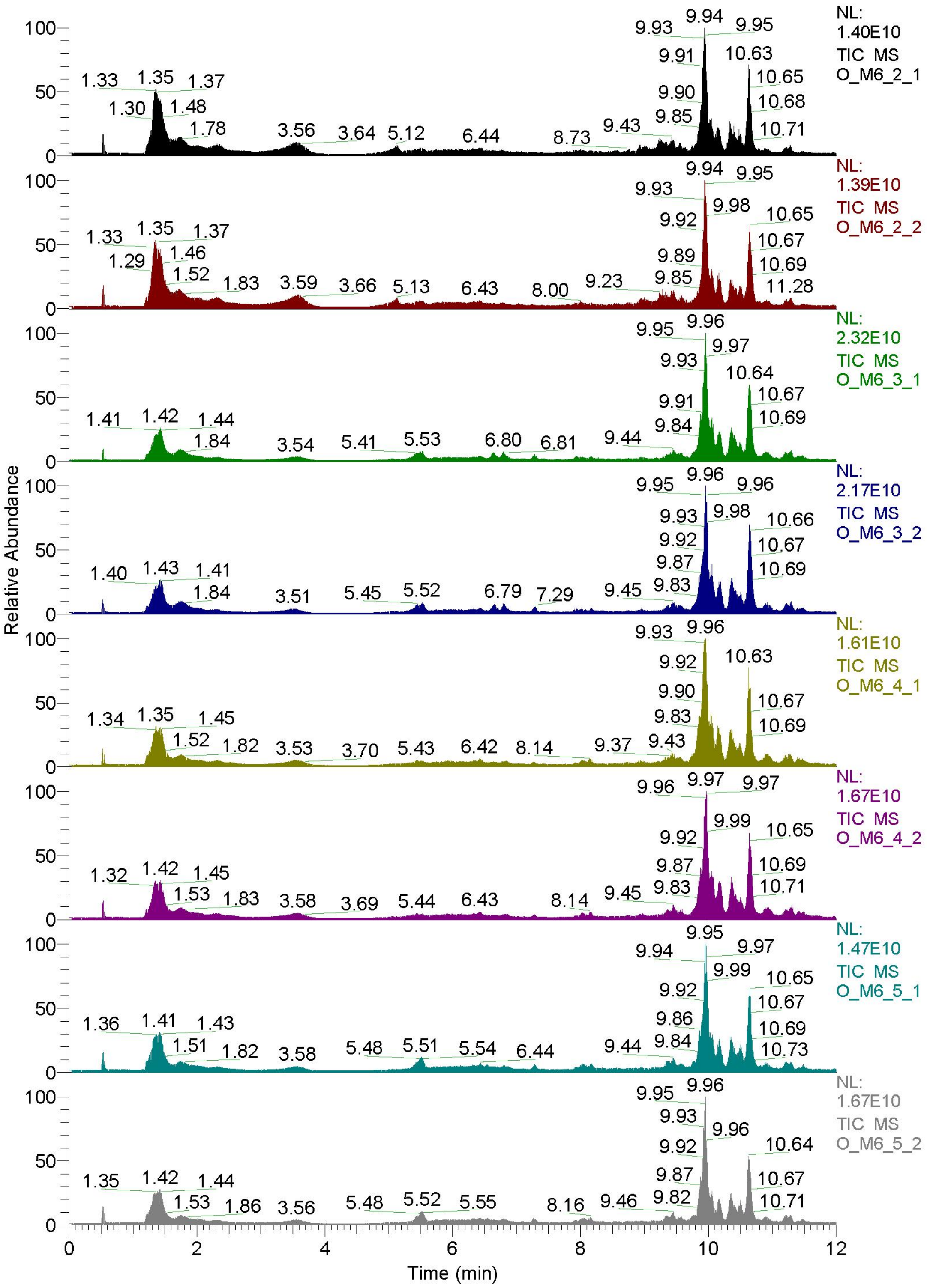

NL: 1.40E10 TIC MS O_M6_2_1
NL: 1.39E10 TIC MS O_M6_2_2
NL: 2.32E10 TIC MS O_M6_3_1
NL: 2.17E10 TIC MS O_M6_3_2
NL: 1.61E10 TIC MS O_M6_4_1
NL: 1.67E10 TIC MS O_M6_4_2
NL: 1.47E10 TIC MS O_M6_5_1
NL: 1.67E10 TIC MS O_M6_5_2
Relative Abundance
Time (min)

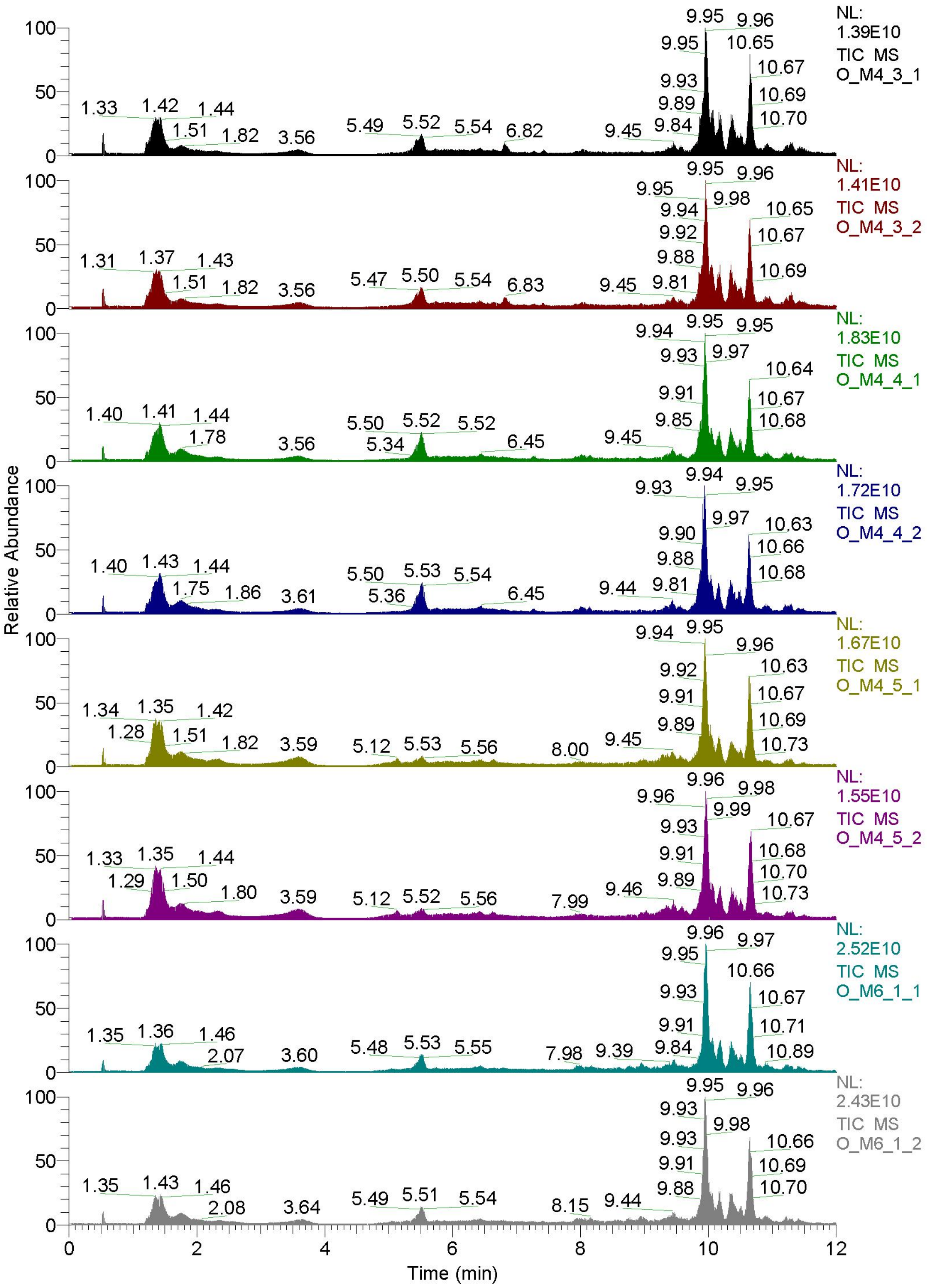

NL: 1.39E10 TIC MS O_M4_3_1
1.33 1.42 1.44 1.51 1.82 3.56 5.49 5.52 5.54 6.82 9.45 9.84 9.89 9.93 9.95 9.95 9.96 10.65 10.67 10.69 10.70
NL: 1.41E10 TIC MS O_M4_3_2
1.31 1.37 1.43 1.51 1.82 3.56 5.47 5.50 5.54 6.83 9.45 9.81 9.88 9.92 9.94 9.95 9.95 9.96 9.98 10.65 10.67 10.69
NL: 1.83E10 TIC MS O_M4_4_1
1.40 1.41 1.44 1.78 3.56 5.50 5.34 5.52 5.52 6.45 9.45 9.85 9.91 9.93 9.94 9.95 9.95 9.97 10.64 10.67 10.68
NL: 1.72E10 TIC MS O_M4_4_2
1.40 1.43 1.44 1.75 1.86 3.61 5.50 5.36 5.53 5.54 6.45 9.44 9.81 9.88 9.90 9.93 9.94 9.95 9.97 10.63 10.66 10.68
NL: 1.67E10 TIC MS O_M4_5_1
1.34 1.28 1.35 1.42 1.51 1.82 3.59 5.12 5.53 5.56 8.00 9.45 9.89 9.91 9.92 9.94 9.95 9.96 10.63 10.67 10.69 10.73
NL: 1.55E10 TIC MS O_M4_5_2
1.33 1.29 1.35 1.44 1.50 1.80 3.59 5.12 5.52 5.56 7.99 9.46 9.89 9.91 9.93 9.96 9.96 9.98 9.99 10.67 10.68 10.70 10.73
NL: 2.52E10 TIC MS O_M6_1_1
1.35 1.36 1.46 2.07 3.60 5.48 5.53 5.55 7.98 9.39 9.84 9.91 9.93 9.95 9.96 9.97 10.66 10.67 10.71 10.89
NL: 2.43E10 TIC MS O_M6_1_2
1.35 1.43 1.46 2.08 3.64 5.49 5.51 5.54 8.15 9.44 9.88 9.91 9.93 9.93 9.95 9.96 9.98 10.66 10.69 10.70
Relative Abundance
0 50 100
Time (min)
0 2 4 6 8 10 12

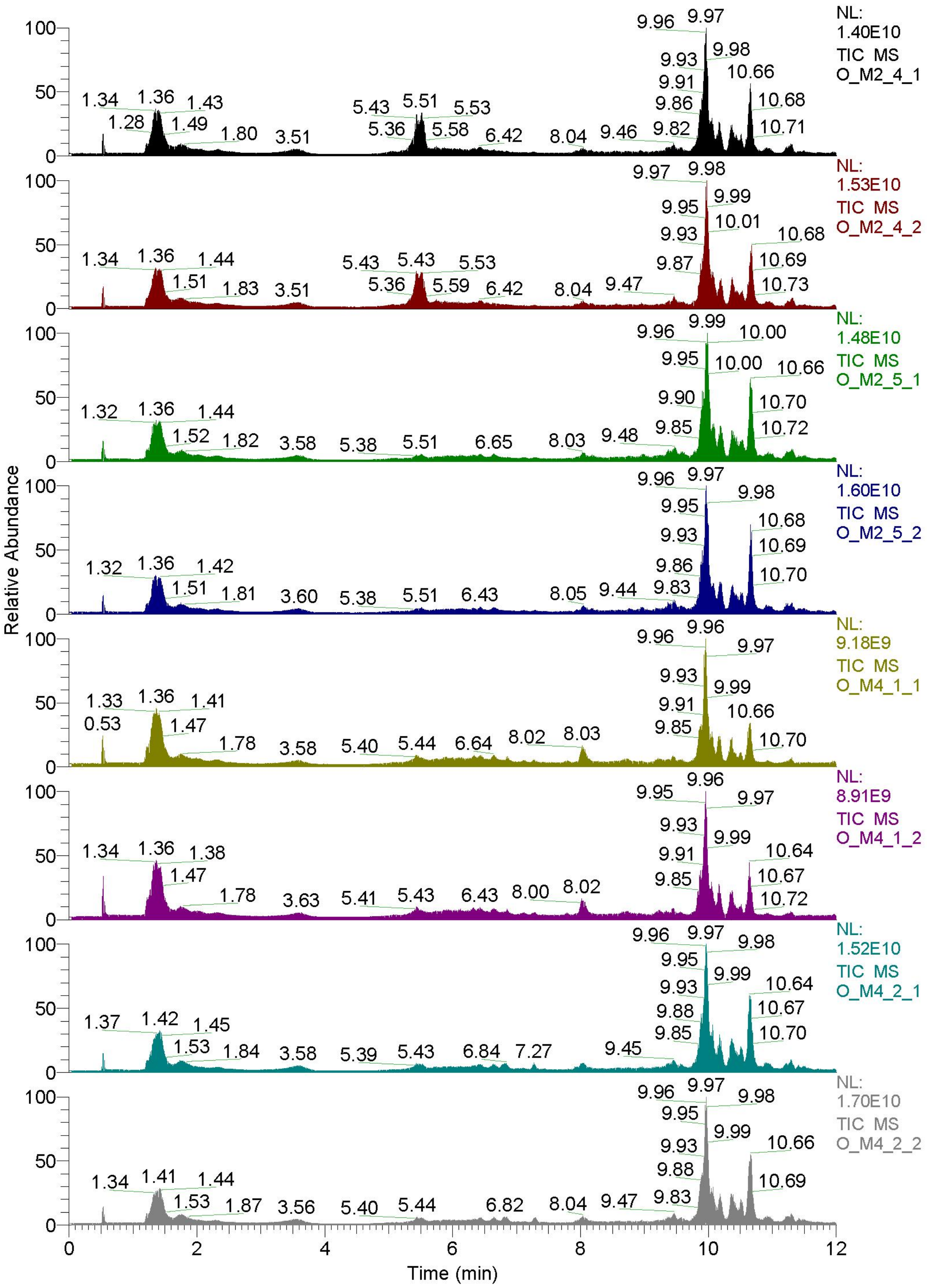

NL: 1.40E10
TIC MS
O_M2_4_1
9.96 9.97 9.98 9.93 9.91 9.86 9.82 10.66 10.68 10.71
1.34 1.36 1.43 1.28 1.49 1.80 3.51 5.43 5.51 5.53 5.36 5.58 6.42 8.04 9.46
NL: 1.53E10
TIC MS
O_M2_4_2
9.97 9.98 9.99 9.95 10.01 9.93 9.87 10.68 10.69 10.73
1.34 1.36 1.44 1.51 1.83 3.51 5.43 5.43 5.53 5.36 5.59 6.42 8.04 9.47
NL: 1.48E10
TIC MS
O_M2_5_1
9.96 9.99 10.00 9.95 10.00 10.66 9.90 9.85 10.70 10.72
1.32 1.36 1.44 1.52 1.82 3.58 5.38 5.51 6.65 8.03 9.48
NL: 1.60E10
TIC MS
O_M2_5_2
9.96 9.97 9.98 9.95 10.68 9.93 10.69 9.86 9.83 10.70
1.32 1.36 1.42 1.51 1.81 3.60 5.38 5.51 6.43 8.05 9.44
NL: 9.18E9
TIC MS
O_M4_1_1
9.96 9.96 9.97 9.93 9.99 9.91 10.66 9.85 10.70
1.33 1.36 1.41 0.53 1.47 1.78 3.58 5.40 5.44 6.64 8.02 8.03
NL: 8.91E9
TIC MS
O_M4_1_2
9.95 9.96 9.97 9.93 9.99 9.91 10.64 9.85 10.67 10.72
1.34 1.36 1.38 1.47 1.78 3.63 5.41 5.43 6.43 8.00 8.02
NL: 1.52E10
TIC MS
O_M4_2_1
9.96 9.97 9.98 9.95 9.99 9.93 10.64 9.88 10.67 9.85 10.70
1.37 1.42 1.45 1.53 1.84 3.58 5.39 5.43 6.84 7.27 9.45
NL: 1.70E10
TIC MS
O_M4_2_2
9.96 9.97 9.98 9.95 9.99 9.93 10.66 9.88 10.69 9.83
1.34 1.41 1.44 1.53 1.87 3.56 5.40 5.44 6.82 8.04 9.47
Relative Abundance
100
50
0
0 2 4 6 8 10 12
Time (min)

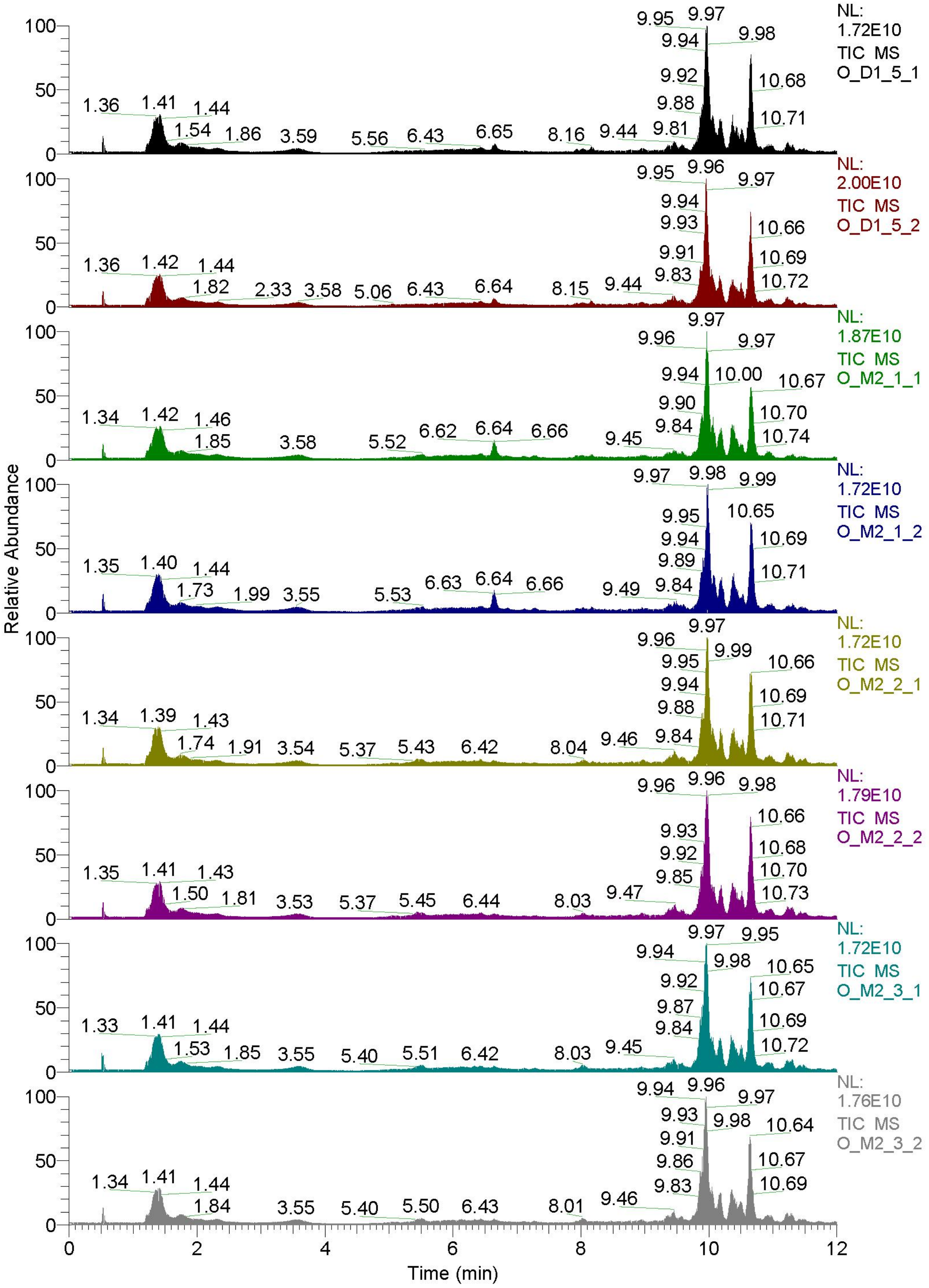

Relative Abundance
Time (min)
0 2 4 6 8 10 12
100 50 0
NL: 1.72E10 TIC MS O_D1_5_1
1.36 1.41 1.44 1.54 1.86 3.59 5.56 6.43 6.65 8.16 9.44 9.81 9.88 9.92 9.94 9.95 9.97 9.98 10.68 10.71
NL: 2.00E10 TIC MS O_D1_5_2
1.36 1.42 1.44 1.82 2.33 3.58 5.06 6.43 6.64 8.15 9.44 9.83 9.91 9.93 9.94 9.95 9.96 9.97 10.66 10.69 10.72
NL: 1.87E10 TIC MS O_M2_1_1
1.34 1.42 1.46 1.85 3.58 5.52 6.62 6.64 6.66 9.45 9.84 9.90 9.94 9.96 9.97 9.97 10.00 10.67 10.70 10.74
NL: 1.72E10 TIC MS O_M2_1_2
1.35 1.40 1.44 1.73 1.99 3.55 5.53 6.63 6.64 6.66 9.49 9.84 9.89 9.94 9.95 9.97 9.98 9.99 10.65 10.69 10.71
NL: 1.72E10 TIC MS O_M2_2_1
1.34 1.39 1.43 1.74 1.91 3.54 5.37 5.43 6.42 8.04 9.46 9.84 9.88 9.94 9.95 9.96 9.97 9.99 10.66 10.69 10.71
NL: 1.79E10 TIC MS O_M2_2_2
1.35 1.41 1.43 1.50 1.81 3.53 5.37 5.45 6.44 8.03 9.47 9.85 9.92 9.93 9.96 9.96 9.98 10.66 10.68 10.70 10.73
NL: 1.72E10 TIC MS O_M2_3_1
1.33 1.41 1.44 1.53 1.85 3.55 5.40 5.51 6.42 8.03 9.45 9.84 9.87 9.92 9.94 9.97 9.95 9.98 10.65 10.67 10.69 10.72
NL: 1.76E10 TIC MS O_M2_3_2
1.34 1.41 1.44 1.84 3.55 5.40 5.50 6.43 8.01 9.46 9.83 9.86 9.91 9.93 9.94 9.96 9.97 9.98 10.64 10.67 10.69

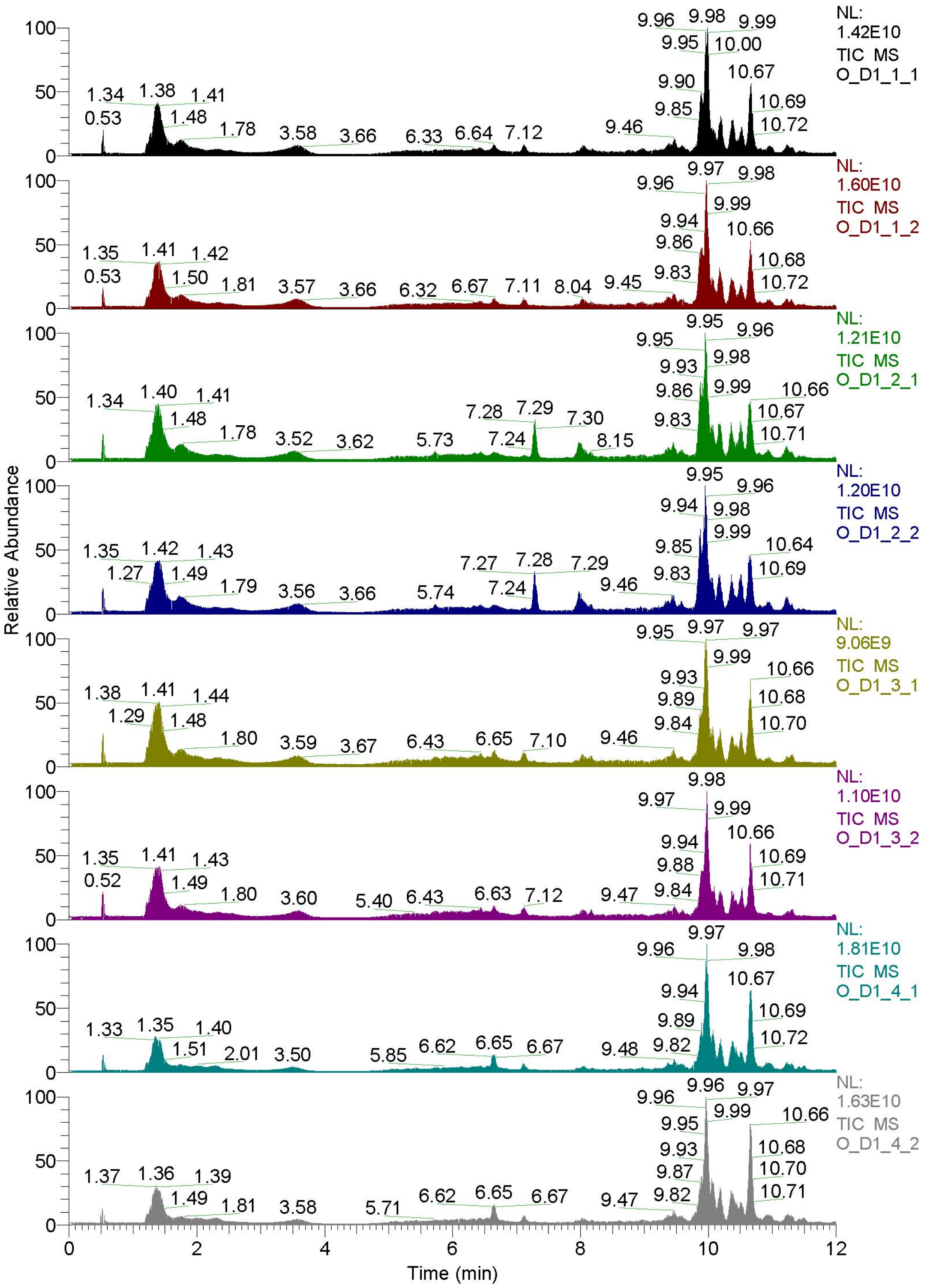

NL: 1.42E10 TIC MS O_D1_1_1
NL: 1.60E10 TIC MS O_D1_1_2
NL: 1.21E10 TIC MS O_D1_2_1
NL: 1.20E10 TIC MS O_D1_2_2
NL: 9.06E9 TIC MS O_D1_3_1
NL: 1.10E10 TIC MS O_D1_3_2
NL: 1.81E10 TIC MS O_D1_4_1
NL: 1.63E10 TIC MS O_D1_4_2
Relative Abundance
Time (min)

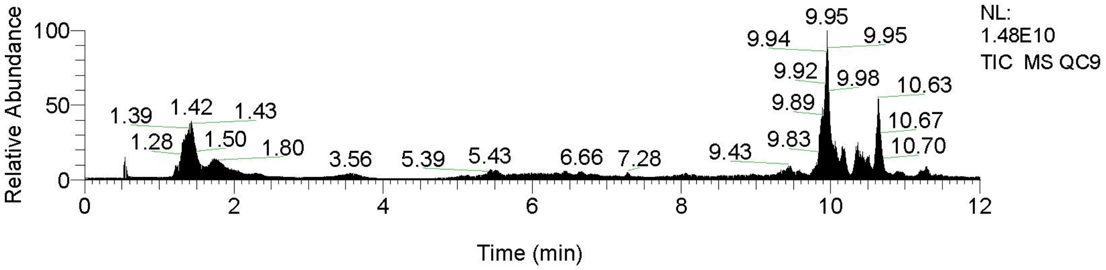

Supplementary Figure 4 The total ion chromatograms (TIC) of each ovarian tissue sample captured in negative ion mode.

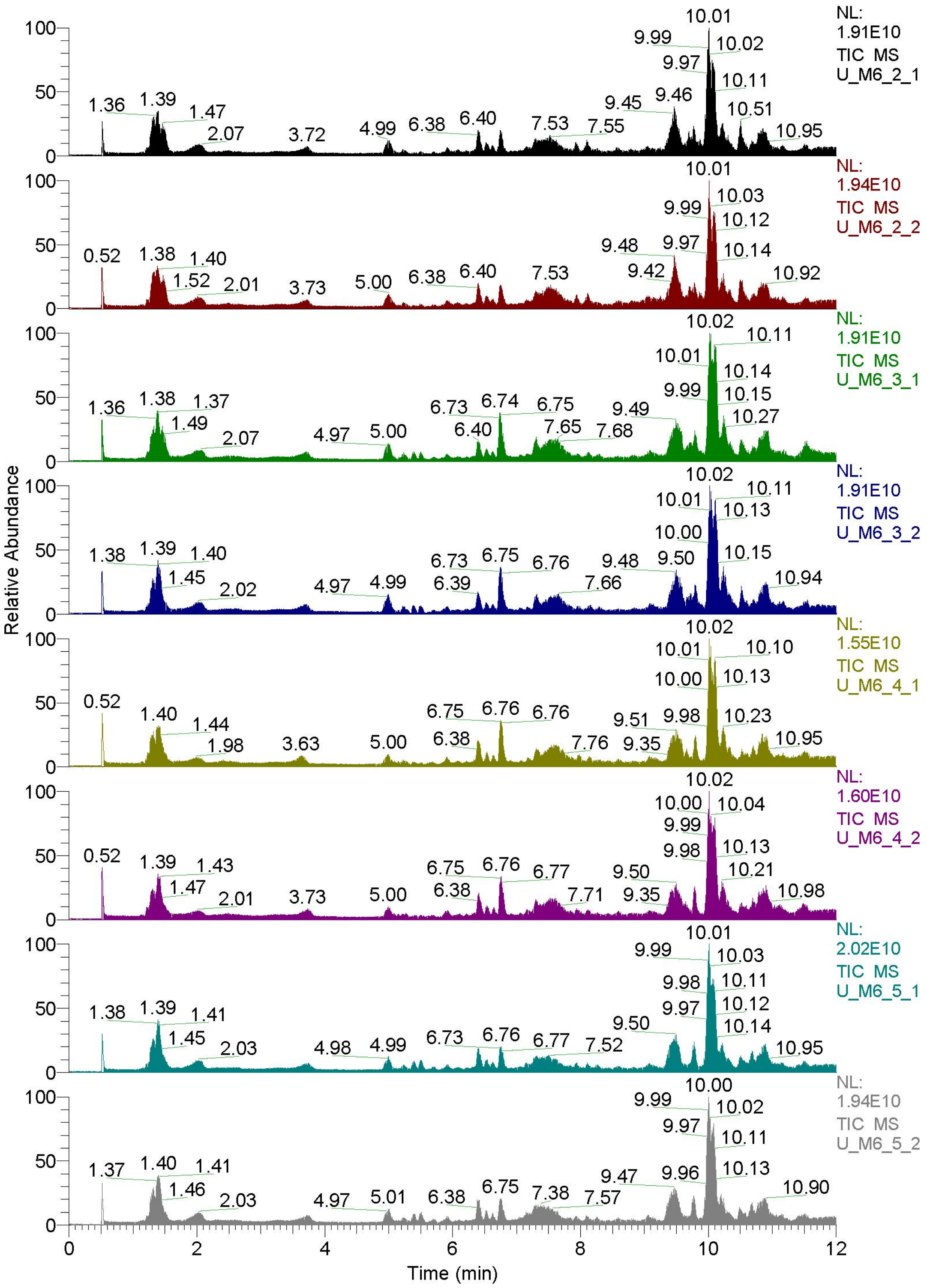

NL: 1.91E10 TIC MS U_M6_2_1
1.36 1.39 1.47 2.07 3.72 4.99 6.38 6.40 7.53 7.55 9.45 9.46 9.99 10.01 10.02 9.97 10.11 10.51 10.95
NL: 1.94E10 TIC MS U_M6_2_2
0.52 1.38 1.40 1.52 2.01 3.73 5.00 6.38 6.40 7.53 9.48 9.42 9.99 10.01 10.03 10.12 9.97 10.14 10.92
NL: 1.91E10 TIC MS U_M6_3_1
1.36 1.38 1.37 1.49 2.07 4.97 5.00 6.73 6.74 6.75 6.40 7.65 7.68 9.49 9.99 10.01 10.02 10.11 10.14 10.15 10.27
NL: 1.91E10 TIC MS U_M6_3_2
1.38 1.39 1.40 1.45 2.02 4.97 4.99 6.73 6.75 6.76 6.39 7.66 9.48 9.50 10.00 10.01 10.02 10.11 10.13 10.15 10.94
NL: 1.55E10 TIC MS U_M6_4_1
0.52 1.40 1.44 1.98 3.63 5.00 6.75 6.76 6.76 6.38 7.76 9.51 9.35 9.98 10.00 10.01 10.02 10.10 10.13 10.23 10.95
NL: 1.60E10 TIC MS U_M6_4_2
0.52 1.39 1.43 1.47 2.01 3.73 5.00 6.75 6.76 6.77 6.38 7.71 9.50 9.35 9.98 9.99 10.00 10.02 10.04 10.13 10.21 10.98
NL: 2.02E10 TIC MS U_M6_5_1
1.38 1.39 1.41 1.45 2.03 4.98 4.99 6.73 6.76 6.77 7.52 9.50 9.97 9.98 9.99 10.01 10.03 10.11 10.12 10.14 10.95
NL: 1.94E10 TIC MS U_M6_5_2
1.37 1.40 1.41 1.46 2.03 4.97 5.01 6.38 6.75 7.38 7.57 9.47 9.96 9.97 9.99 10.00 10.02 10.11 10.13 10.90
Relative Abundance
100
50
0
Time (min)
0 2 4 6 8 10 12

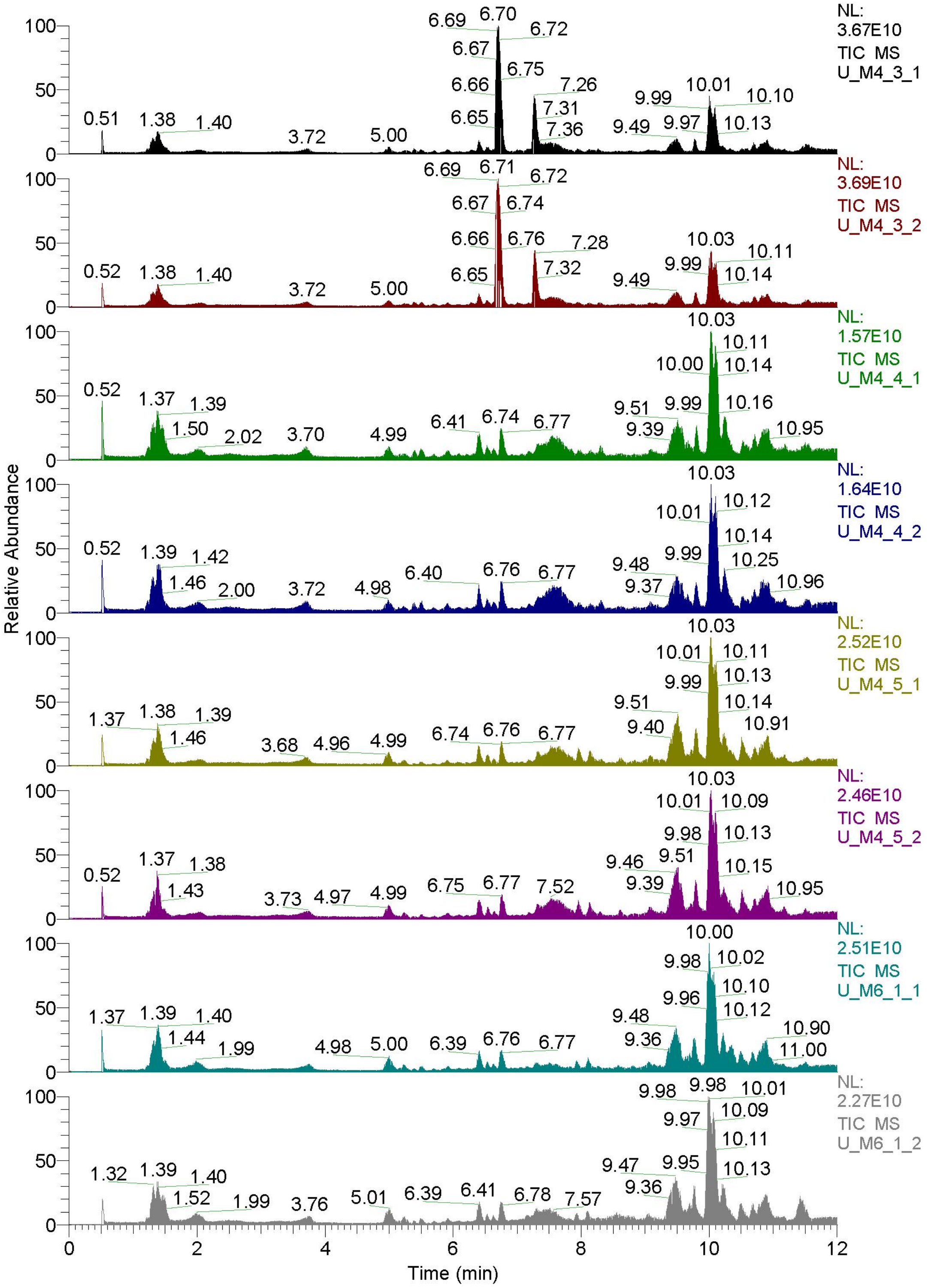
Relative Abundance
Time (min)
NL: 3.67E10 TIC MS U_M4_3_1
NL: 3.69E10 TIC MS U_M4_3_2
NL: 1.57E10 TIC MS U_M4_4_1
NL: 1.64E10 TIC MS U_M4_4_2
NL: 2.52E10 TIC MS U_M4_5_1
NL: 2.46E10 TIC MS U_M4_5_2
NL: 2.51E10 TIC MS U_M6_1_1
NL: 2.27E10 TIC MS U_M6_1_2

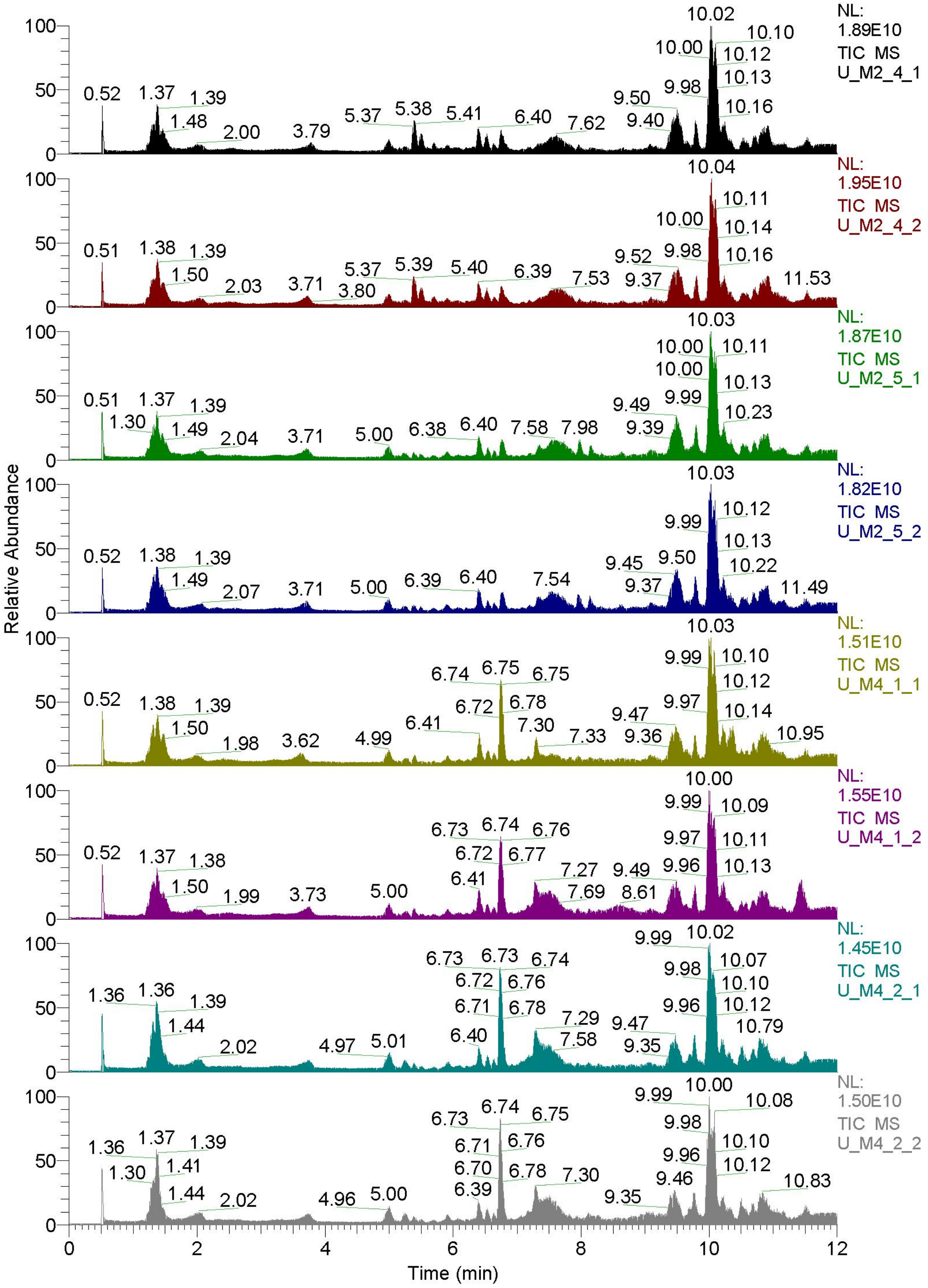

NL: 1.89E10 TIC MS U_M2_4_1
10.02 10.10 10.00 10.12 10.13 9.98 9.50 10.16 9.40 0.52 1.37 1.39 1.48 2.00 3.79 5.37 5.38 5.41 6.40 7.62
NL: 1.95E10 TIC MS U_M2_4_2
10.04 10.11 10.00 10.14 9.98 9.52 10.16 9.37 11.53 0.51 1.38 1.39 1.50 2.03 3.71 3.80 5.37 5.39 5.40 6.39 7.53
NL: 1.87E10 TIC MS U_M2_5_1
10.03 10.00 10.11 10.00 10.13 9.99 9.49 10.23 9.39 0.51 1.37 1.39 1.30 1.49 2.04 3.71 5.00 6.38 6.40 7.58 7.98
NL: 1.82E10 TIC MS U_M2_5_2
10.03 10.12 9.99 10.13 9.45 9.50 10.22 9.37 11.49 0.52 1.38 1.39 1.49 2.07 3.71 5.00 6.39 6.40 7.54
NL: 1.51E10 TIC MS U_M4_1_1
10.03 9.99 10.10 10.12 9.97 9.47 10.14 9.36 10.95 0.52 1.38 1.39 1.50 1.98 3.62 4.99 6.41 6.74 6.75 6.75 6.72 6.78 7.30 7.33
NL: 1.55E10 TIC MS U_M4_1_2
10.00 9.99 10.09 9.97 10.11 9.49 9.96 10.13 8.61 0.52 1.37 1.38 1.50 1.99 3.73 5.00 6.41 6.73 6.74 6.76 6.72 6.77 7.27 7.69
NL: 1.45E10 TIC MS U_M4_2_1
9.99 10.02 10.07 9.98 10.10 9.96 10.12 9.47 10.79 9.35 1.36 1.36 1.39 1.44 2.02 4.97 5.01 6.40 6.73 6.73 6.74 6.72 6.76 6.71 6.78 7.29 7.58
NL: 1.50E10 TIC MS U_M4_2_2
9.99 10.00 10.08 9.98 10.10 9.96 10.12 9.46 10.83 9.35 1.36 1.37 1.39 1.30 1.41 1.44 2.02 4.96 5.00 6.39 6.73 6.74 6.75 6.71 6.76 6.70 6.78 7.30
100 50 0
Relative Abundance
0 2 4 6 8 10 12
Time (min)

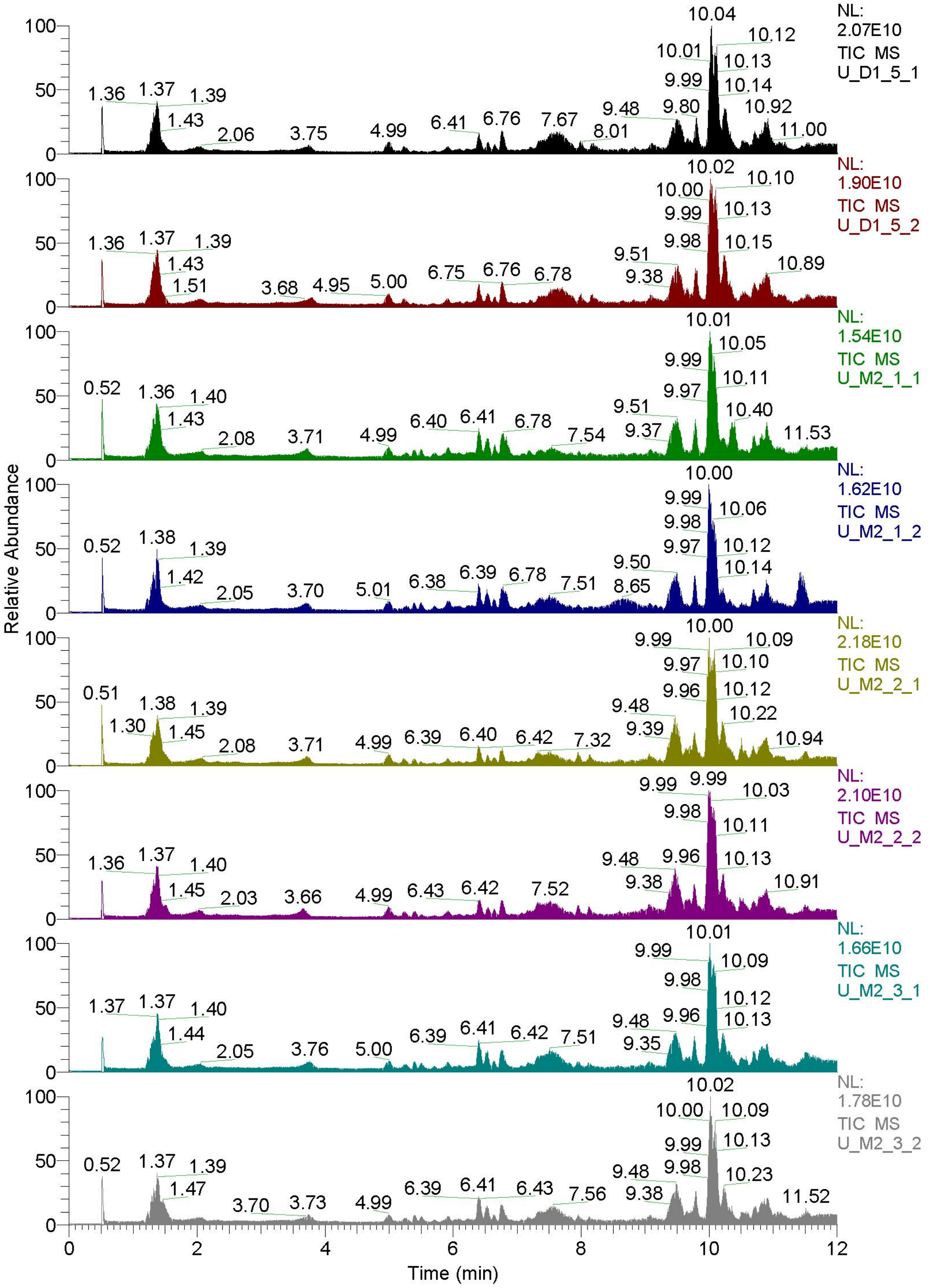

NL: 2.07E10 TIC MS U_D1_5_1
10.04 10.12 10.01 10.13 9.99 10.14 1.36 1.37 1.39 9.48 9.80 10.92 1.43 2.06 3.75 4.99 6.41 6.76 7.67 8.01 11.00
NL: 1.90E10 TIC MS U_D1_5_2
10.02 10.10 10.00 9.99 10.13 1.36 1.37 1.39 9.98 10.15 1.43 9.51 10.89 1.51 3.68 4.95 5.00 6.75 6.76 6.78 9.38
NL: 1.54E10 TIC MS U_M2_1_1
10.01 10.05 9.99 10.11 0.52 1.36 1.40 9.97 9.51 10.40 1.43 6.40 6.41 6.78 2.08 3.71 4.99 7.54 9.37 11.53
NL: 1.62E10 TIC MS U_M2_1_2
10.00 9.99 10.06 9.98 0.52 1.38 1.39 9.97 10.12 9.50 10.14 1.42 6.38 6.39 6.78 2.05 3.70 5.01 7.51 8.65
NL: 2.18E10 TIC MS U_M2_2_1
10.00 9.99 10.09 9.97 10.10 0.51 1.38 1.39 9.96 10.12 9.48 1.30 1.45 10.22 9.39 6.39 6.40 6.42 7.32 2.08 3.71 4.99 10.94
NL: 2.10E10 TIC MS U_M2_2_2
9.99 9.99 10.03 9.98 10.11 1.36 1.37 1.40 9.48 9.96 10.13 1.45 2.03 3.66 4.99 6.43 6.42 7.52 9.38 10.91
NL: 1.66E10 TIC MS U_M2_3_1
10.01 9.99 10.09 9.98 1.37 1.37 1.40 10.12 9.48 9.96 10.13 1.44 6.39 6.41 6.42 7.51 2.05 3.76 5.00 9.35
NL: 1.78E10 TIC MS U_M2_3_2
10.02 10.00 10.09 9.99 10.13 0.52 1.37 1.39 9.48 9.98 10.23 1.47 3.70 3.73 4.99 6.39 6.41 6.43 7.56 9.38 11.52
Relative Abundance
100
50
0
0 2 4 6 8 10 12
Time (min)

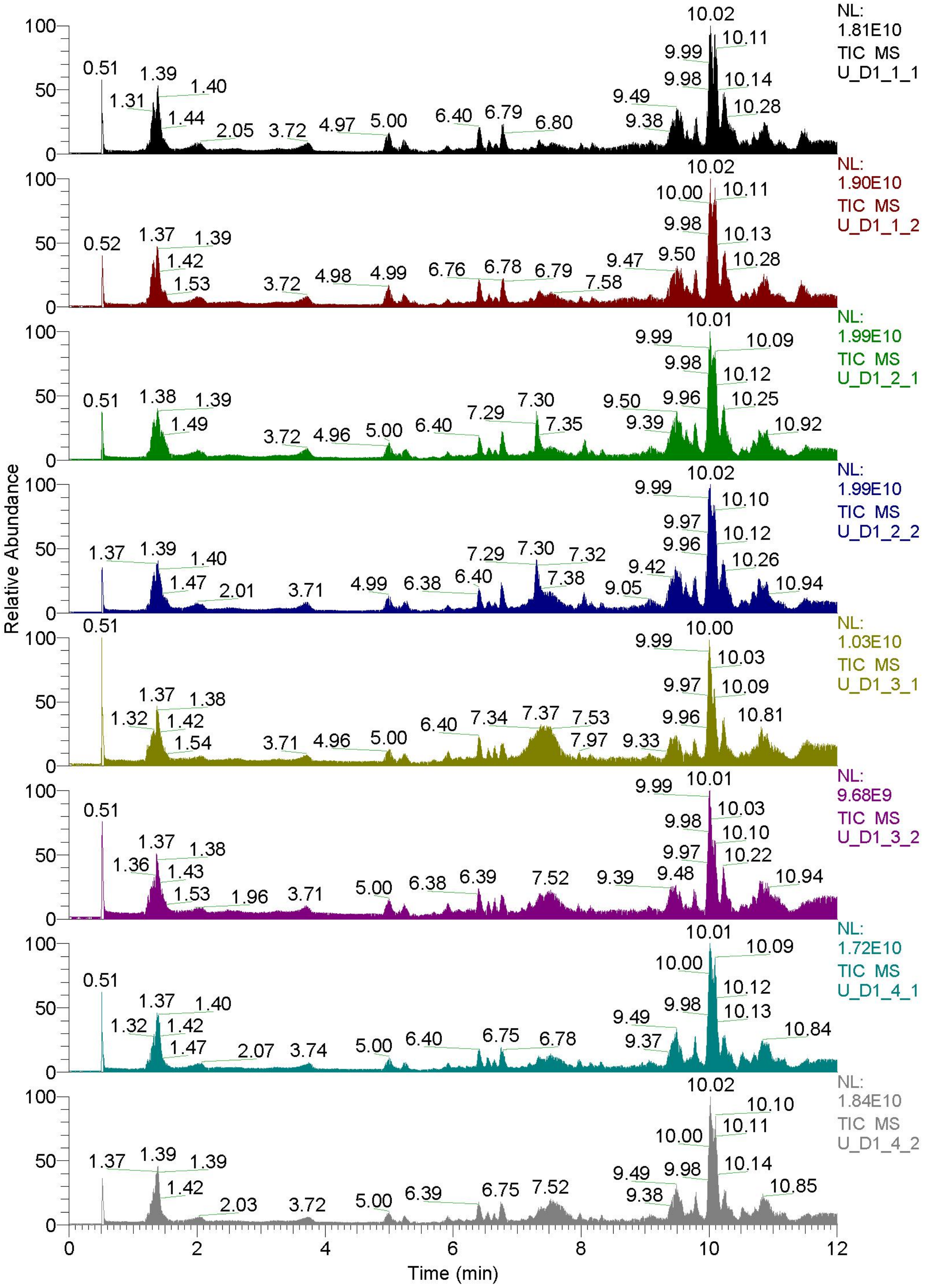

Relative Abundance
Time (min)
0
2
4
6
8
10
12
100
50
0
NL: 1.81E10 TIC MS U_D1_1_1
10.02 10.11 9.99 9.98 10.14 0.51 1.39 1.40 1.31 1.44 2.05 3.72 4.97 5.00 6.40 6.79 6.80 9.49 9.38 10.28
NL: 1.90E10 TIC MS U_D1_1_2
10.02 10.00 10.11 9.98 10.13 0.52 1.37 1.39 1.42 1.53 3.72 4.98 4.99 6.76 6.78 6.79 7.58 9.47 9.50 10.28
NL: 1.99E10 TIC MS U_D1_2_1
10.01 9.99 10.09 9.98 10.12 0.51 1.38 1.39 1.49 3.72 4.96 5.00 6.40 7.29 7.30 7.35 9.50 9.96 9.39 10.25 10.92
NL: 1.99E10 TIC MS U_D1_2_2
10.02 9.99 10.10 9.97 10.12 9.96 1.37 1.39 1.40 1.47 2.01 3.71 4.99 6.38 6.40 7.29 7.30 7.32 7.38 9.42 9.05 10.26 10.94
NL: 1.03E10 TIC MS U_D1_3_1
0.51 10.00 9.99 10.03 9.97 10.09 1.37 1.38 1.32 1.42 1.54 3.71 4.96 5.00 6.40 7.34 7.37 7.53 7.97 9.33 9.96 10.81
NL: 9.68E9 TIC MS U_D1_3_2
9.99 10.01 10.03 9.98 10.10 0.51 1.37 1.38 1.36 1.43 1.53 1.96 3.71 5.00 6.38 6.39 7.52 9.39 9.48 9.97 10.22 10.94
NL: 1.72E10 TIC MS U_D1_4_1
10.01 10.09 10.00 10.12 0.51 1.37 1.40 1.32 1.42 1.47 2.07 3.74 5.00 6.40 6.75 6.78 9.49 9.98 9.37 10.13 10.84
NL: 1.84E10 TIC MS U_D1_4_2
10.02 10.10 10.00 10.11 1.37 1.39 1.39 1.42 2.03 3.72 5.00 6.39 6.75 7.52 9.49 9.98 9.38 10.14 10.85

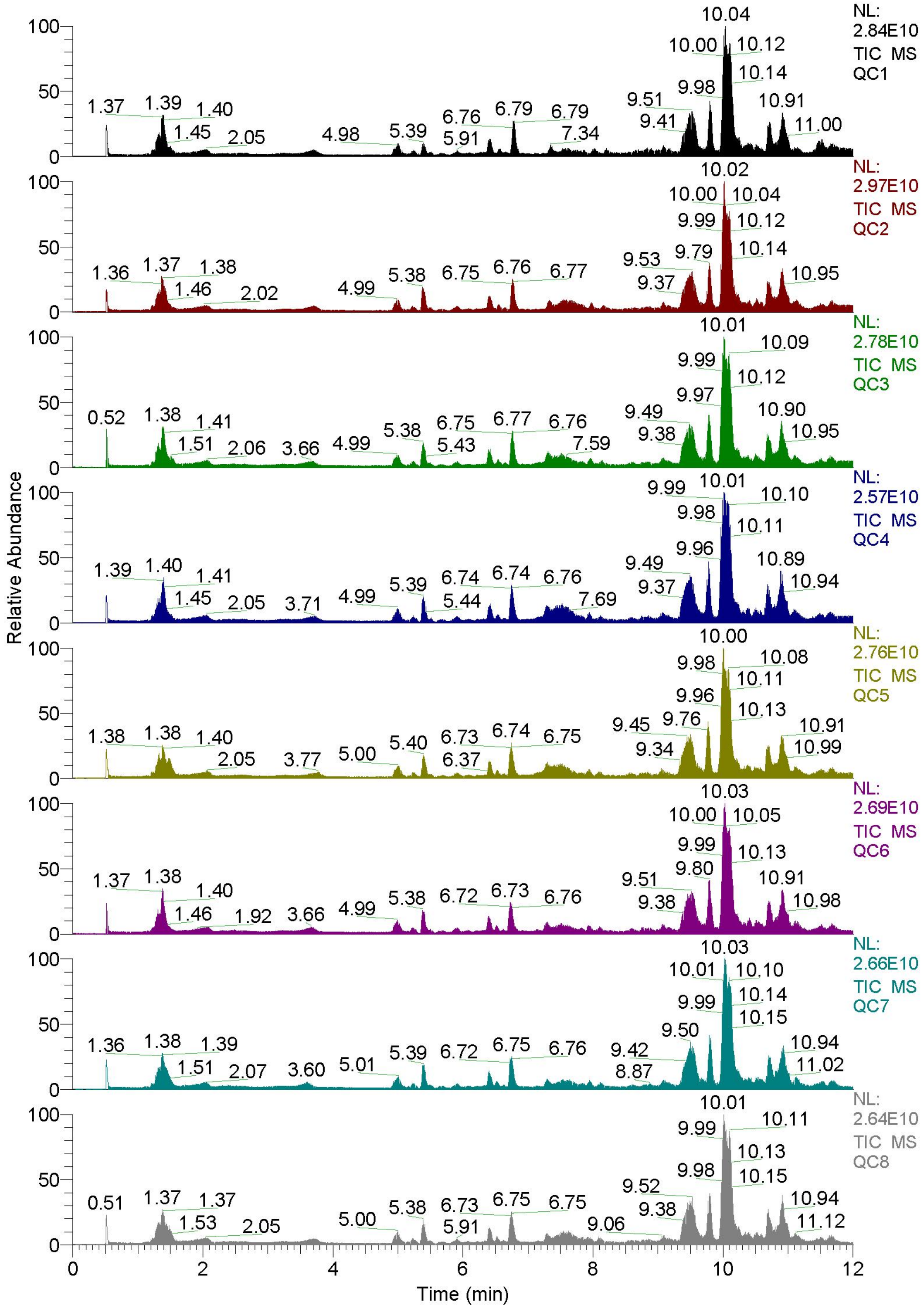

Supplementary Figure 5 The total ion chromatograms (TIC) of each uterine tissue sample captured in positive ion mode.

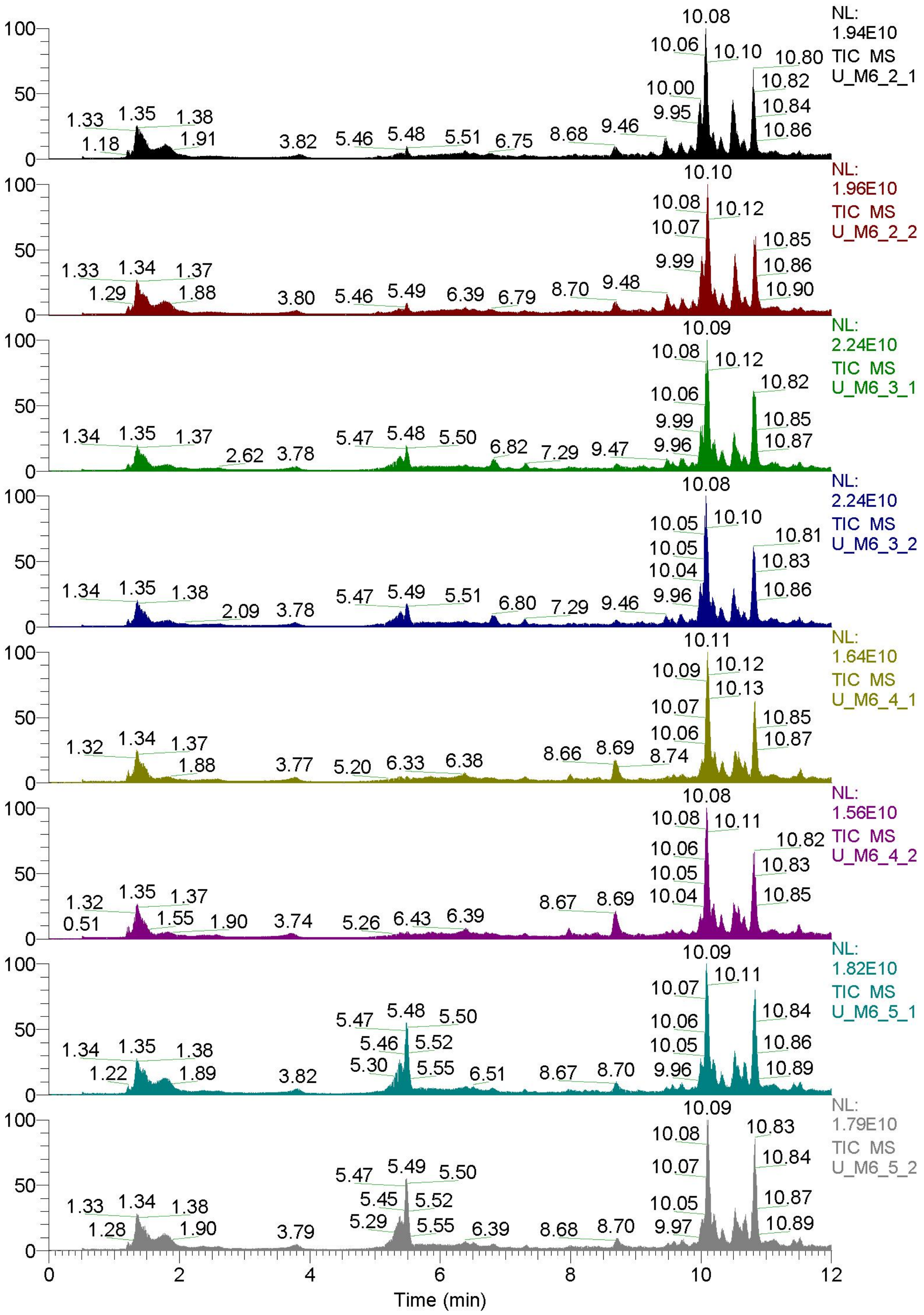
NL: 1.94E10 TIC MS U_M6_2_1
10.08
10.06
10.10
10.80
10.82
10.00
9.95
10.84
10.86
1.33
1.35
1.38
1.18
1.91
3.82
5.46
5.48
5.51
6.75
8.68
9.46
NL: 1.96E10 TIC MS U_M6_2_2
10.10
10.08
10.12
10.07
10.85
9.99
10.86
10.90
1.33
1.34
1.37
1.29
1.88
3.80
5.46
5.49
6.39
6.79
8.70
9.48
NL: 2.24E10 TIC MS U_M6_3_1
10.09
10.08
10.12
10.82
10.06
9.99
10.85
9.96
10.87
1.34
1.35
1.37
2.62
3.78
5.47
5.48
5.50
6.82
7.29
9.47
NL: 2.24E10 TIC MS U_M6_3_2
10.08
10.05
10.10
10.81
10.05
10.83
10.04
9.96
10.86
1.34
1.35
1.38
2.09
3.78
5.47
5.49
5.51
6.80
7.29
9.46
NL: 1.64E10 TIC MS U_M6_4_1
10.11
10.09
10.12
10.13
10.07
10.85
10.06
10.87
8.74
1.32
1.34
1.37
1.88
3.77
5.20
6.33
6.38
8.66
8.69
NL: 1.56E10 TIC MS U_M6_4_2
10.08
10.08
10.11
10.82
10.06
10.83
10.05
10.04
10.85
1.32
1.35
1.37
0.51
1.55
1.90
3.74
5.26
6.43
6.39
8.67
8.69
NL: 1.82E10 TIC MS U_M6_5_1
10.09
10.07
10.11
10.84
10.06
10.86
10.05
9.96
10.89
1.34
1.35
1.38
1.22
1.89
3.82
5.47
5.48
5.50
5.46
5.52
5.30
5.55
6.51
8.67
8.70
NL: 1.79E10 TIC MS U_M6_5_2
10.09
10.08
10.83
10.07
10.84
10.05
10.87
9.97
10.89
1.33
1.34
1.38
1.28
1.90
3.79
5.47
5.49
5.50
5.45
5.52
5.29
5.55
6.39
8.68
8.70
100
50
0
0
2
4
6
8
10
12
Time (min)

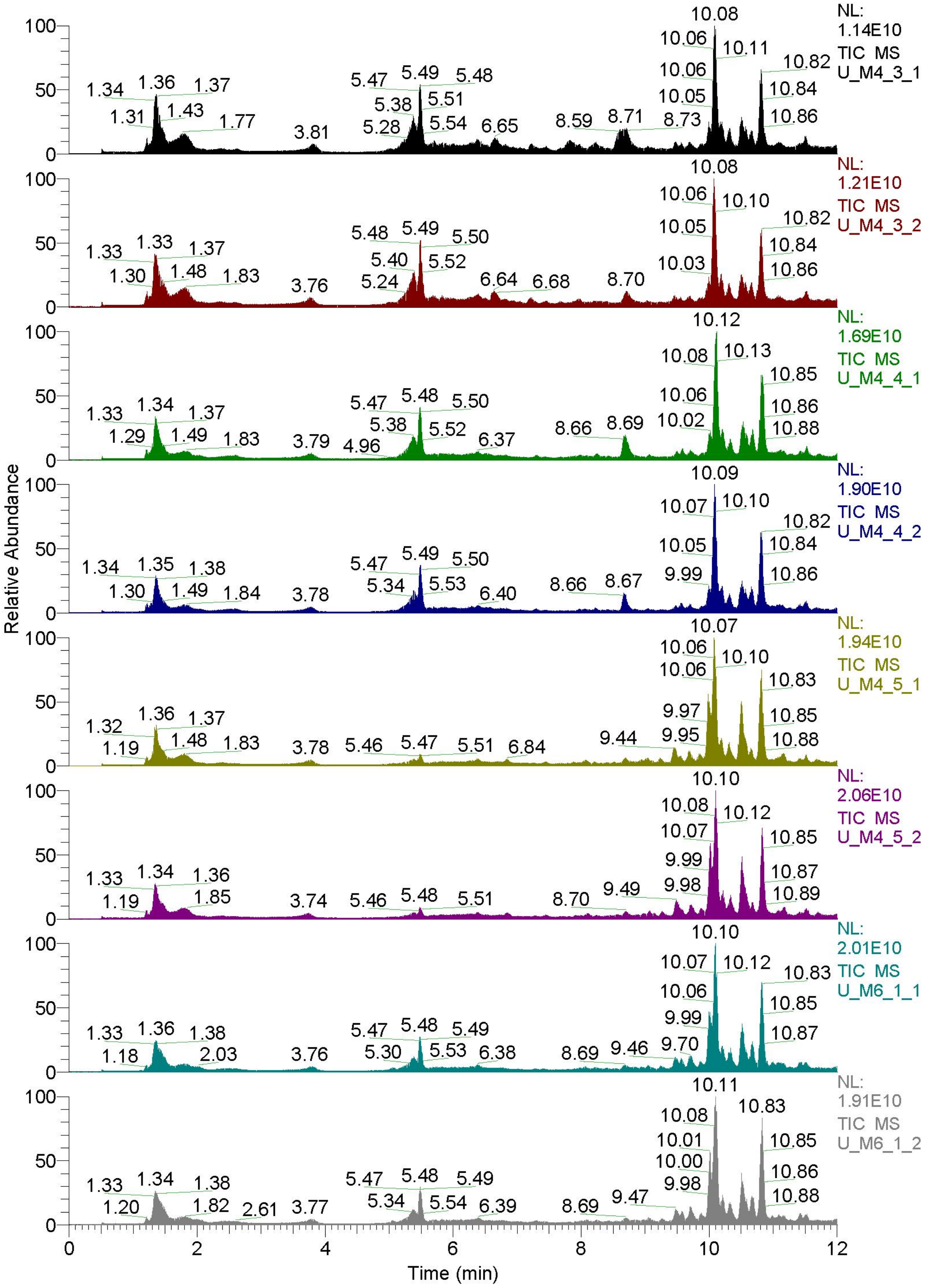
NL: 1.14E10 TIC MS U_M4_3_1
NL: 1.21E10 TIC MS U_M4_3_2
NL: 1.69E10 TIC MS U_M4_4_1
NL: 1.90E10 TIC MS U_M4_4_2
NL: 1.94E10 TIC MS U_M4_5_1
NL: 2.06E10 TIC MS U_M4_5_2
NL: 2.01E10 TIC MS U_M6_1_1
NL: 1.91E10 TIC MS U_M6_1_2
Relative Abundance
Time (min)

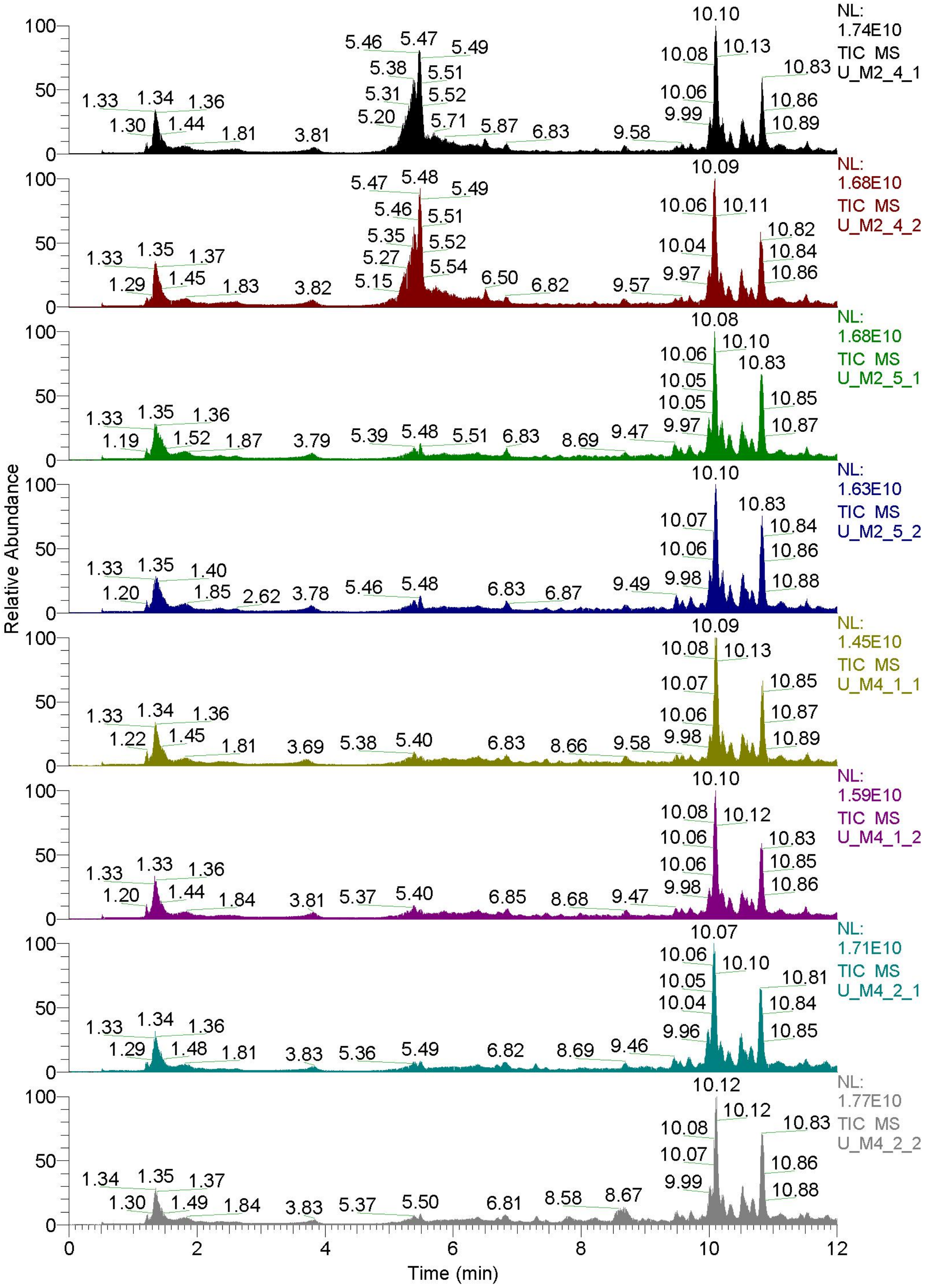

Relative Abundance
Time (min)
0 2 4 6 8 10 12
100 50 0
NL: 1.74E10 TIC MS U_M2_4_1
10.10 10.08 10.13 10.83 10.06 10.86 9.99 10.89 9.58 6.83 5.87 5.71 5.46 5.47 5.49 5.38 5.51 5.31 5.52 5.20 3.81 1.81 1.33 1.34 1.36 1.30 1.44
NL: 1.68E10 TIC MS U_M2_4_2
10.09 10.06 10.11 10.82 10.04 10.84 9.97 10.86 9.57 6.82 6.50 5.47 5.48 5.49 5.46 5.51 5.35 5.52 5.27 5.54 5.15 3.82 1.83 1.33 1.35 1.37 1.29 1.45
NL: 1.68E10 TIC MS U_M2_5_1
10.08 10.10 10.06 10.83 10.05 10.05 10.85 9.97 10.87 9.47 8.69 6.83 5.51 5.48 5.39 3.79 1.87 1.33 1.35 1.36 1.19 1.52
NL: 1.63E10 TIC MS U_M2_5_2
10.10 10.83 10.07 10.84 10.06 10.86 9.98 10.88 9.49 6.87 6.83 5.48 5.46 3.78 2.62 1.85 1.33 1.35 1.40 1.20
NL: 1.45E10 TIC MS U_M4_1_1
10.09 10.08 10.13 10.07 10.85 10.06 10.87 9.98 10.89 9.58 8.66 6.83 5.40 5.38 3.69 1.81 1.33 1.34 1.36 1.22 1.45
NL: 1.59E10 TIC MS U_M4_1_2
10.10 10.08 10.12 10.06 10.83 10.06 10.85 9.98 10.86 9.47 8.68 6.85 5.40 5.37 3.81 1.84 1.33 1.33 1.36 1.20 1.44
NL: 1.71E10 TIC MS U_M4_2_1
10.07 10.06 10.10 10.05 10.81 10.04 10.84 9.96 10.85 9.46 8.69 6.82 5.49 5.36 3.83 1.81 1.33 1.34 1.36 1.29 1.48
NL: 1.77E10 TIC MS U_M4_2_2
10.12 10.12 10.08 10.83 10.07 10.86 9.99 10.88 8.67 8.58 6.81 5.50 5.37 3.83 1.84 1.34 1.35 1.37 1.30 1.49

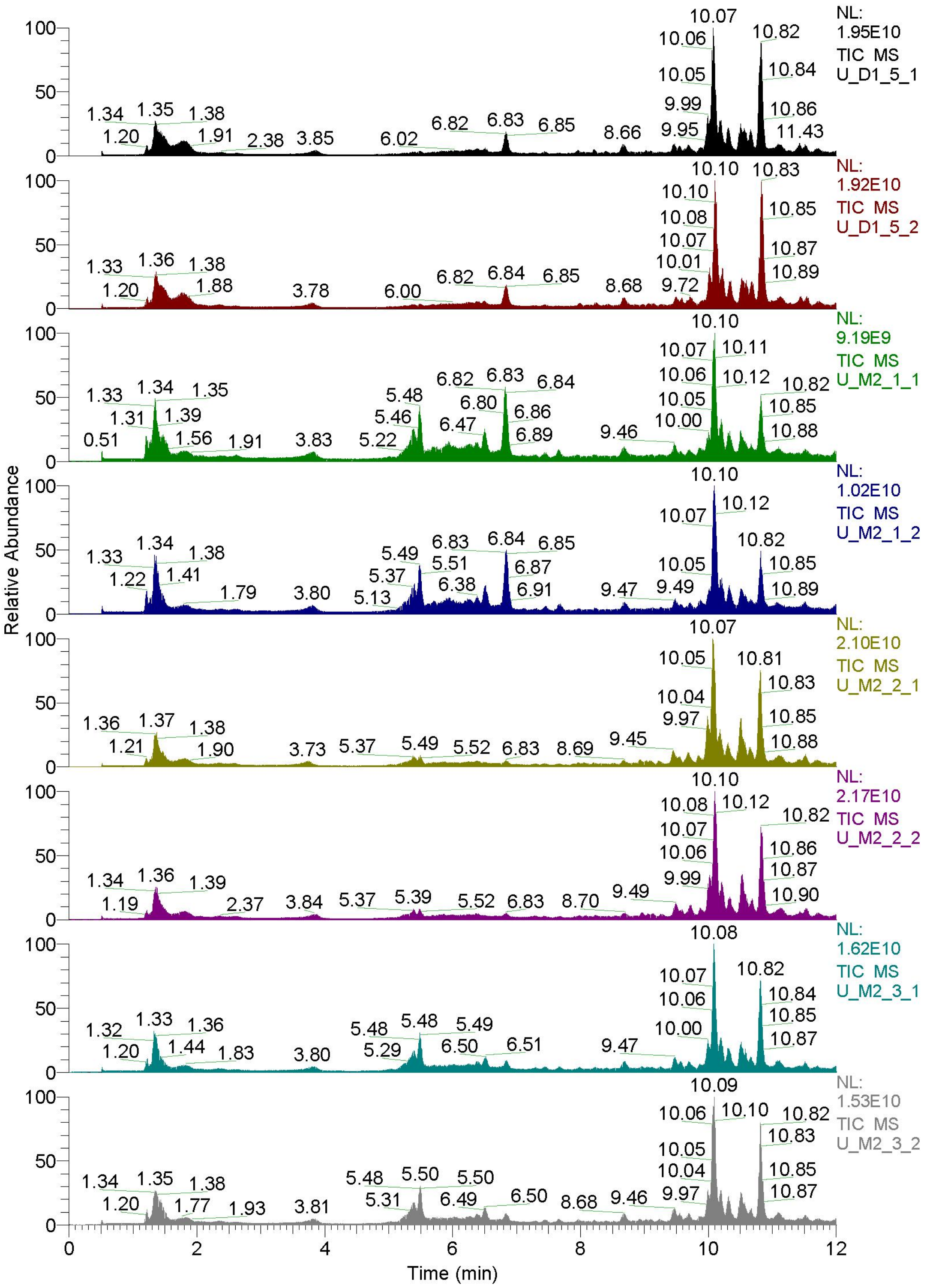

NL: 1.95E10 TIC MS U_D1_5_1
10.07 10.06 10.05 9.99 9.95 10.82 10.84 10.86 11.43 1.34 1.35 1.38 1.20 1.91 2.38 3.85 6.02 6.82 6.83 6.85 8.66
NL: 1.92E10 TIC MS U_D1_5_2
10.10 10.10 10.08 10.07 10.01 9.72 10.83 10.85 10.87 10.89 1.33 1.36 1.38 1.20 1.88 3.78 6.00 6.82 6.84 6.85 8.68
NL: 9.19E9 TIC MS U_M2_1_1
10.10 10.07 10.11 10.06 10.12 10.05 10.00 10.82 10.85 10.88 1.33 1.34 1.35 1.31 1.39 0.51 1.56 1.91 3.83 5.22 5.48 5.46 6.47 6.82 6.83 6.84 6.80 6.86 6.89 9.46
NL: 1.02E10 TIC MS U_M2_1_2
10.10 10.07 10.12 10.82 10.05 10.85 9.49 10.89 1.33 1.34 1.38 1.22 1.41 1.79 3.80 5.13 5.49 5.37 5.51 6.38 6.83 6.84 6.85 6.87 6.91 9.47
NL: 2.10E10 TIC MS U_M2_2_1
10.07 10.05 10.81 10.04 10.83 9.97 10.85 10.88 1.36 1.37 1.38 1.21 1.90 3.73 5.37 5.49 5.52 6.83 8.69 9.45
NL: 2.17E10 TIC MS U_M2_2_2
10.10 10.08 10.12 10.07 10.82 10.06 10.86 9.99 10.87 10.90 1.34 1.36 1.39 1.19 2.37 3.84 5.37 5.39 5.52 6.83 8.70 9.49
NL: 1.62E10 TIC MS U_M2_3_1
10.08 10.07 10.82 10.06 10.84 10.85 10.00 10.87 1.32 1.33 1.36 1.20 1.44 1.83 3.80 5.48 5.48 5.49 5.29 6.50 6.51 9.47
NL: 1.53E10 TIC MS U_M2_3_2
10.09 10.06 10.10 10.82 10.83 10.05 10.04 10.85 9.97 10.87 1.34 1.35 1.38 1.20 1.77 1.93 3.81 5.48 5.50 5.50 5.31 6.49 6.50 8.68 9.46
100 50 0
Relative Abundance
0 2 4 6 8 10 12
Time (min)

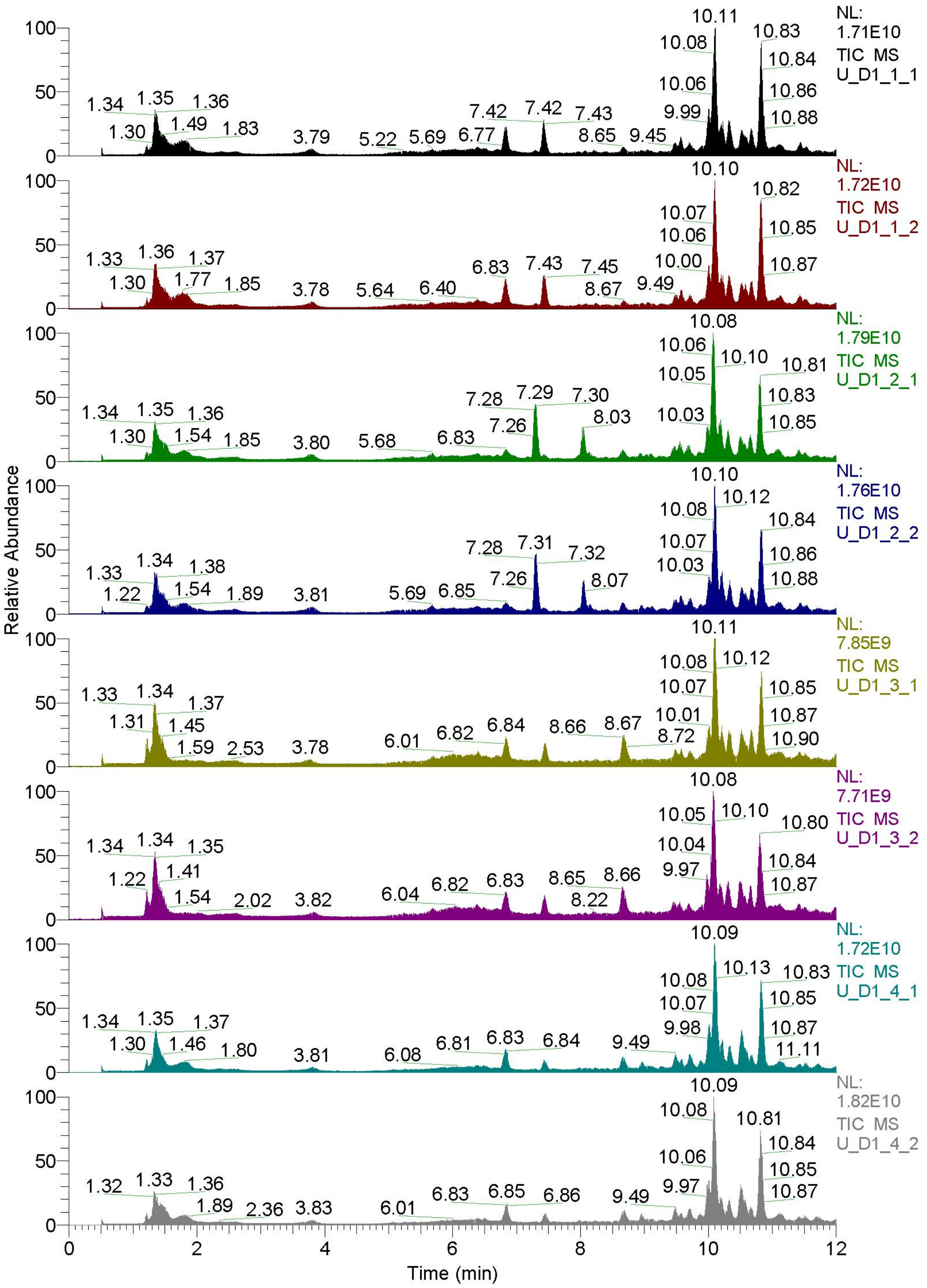

Relative Abundance
Time (min)
NL: 1.71E10 TIC MS U_D1_1_1
10.11 10.08 10.06 9.99 9.45 8.65 7.43 7.42 7.42 6.77 5.69 5.22 3.79 1.83 1.49 1.36 1.35 1.34 1.30 10.83 10.84 10.86 10.88
NL: 1.72E10 TIC MS U_D1_1_2
10.10 10.07 10.06 10.00 9.49 8.67 7.45 7.43 6.83 6.40 5.64 3.78 1.85 1.77 1.37 1.36 1.33 1.30 10.82 10.85 10.87
NL: 1.79E10 TIC MS U_D1_2_1
10.08 10.06 10.05 10.03 10.10 10.81 10.83 10.85 8.03 7.30 7.29 7.28 7.26 6.83 5.68 3.80 1.85 1.54 1.36 1.35 1.34 1.30
NL: 1.76E10 TIC MS U_D1_2_2
10.10 10.08 10.07 10.03 10.12 10.84 10.86 10.88 8.07 7.32 7.31 7.28 7.26 6.85 5.69 3.81 1.89 1.54 1.38 1.34 1.33 1.22
NL: 7.85E9 TIC MS U_D1_3_1
10.11 10.08 10.07 10.01 10.12 10.85 10.87 10.90 8.72 8.67 8.66 6.84 6.82 6.01 3.78 2.53 1.59 1.45 1.37 1.34 1.33 1.31
NL: 7.71E9 TIC MS U_D1_3_2
10.08 10.05 10.04 9.97 10.10 10.80 10.84 10.87 8.66 8.65 8.22 6.83 6.82 6.04 3.82 2.02 1.54 1.41 1.35 1.34 1.34 1.22
NL: 1.72E10 TIC MS U_D1_4_1
10.09 10.08 10.07 9.98 10.13 10.83 10.85 10.87 11.11 9.49 6.84 6.83 6.81 6.08 3.81 1.80 1.46 1.37 1.35 1.34 1.30
NL: 1.82E10 TIC MS U_D1_4_2
10.09 10.08 10.06 9.97 10.81 10.84 10.85 10.87 9.49 6.86 6.85 6.83 6.01 3.83 2.36 1.89 1.36 1.33 1.32
0 2 4 6 8 10 12
0 50 100

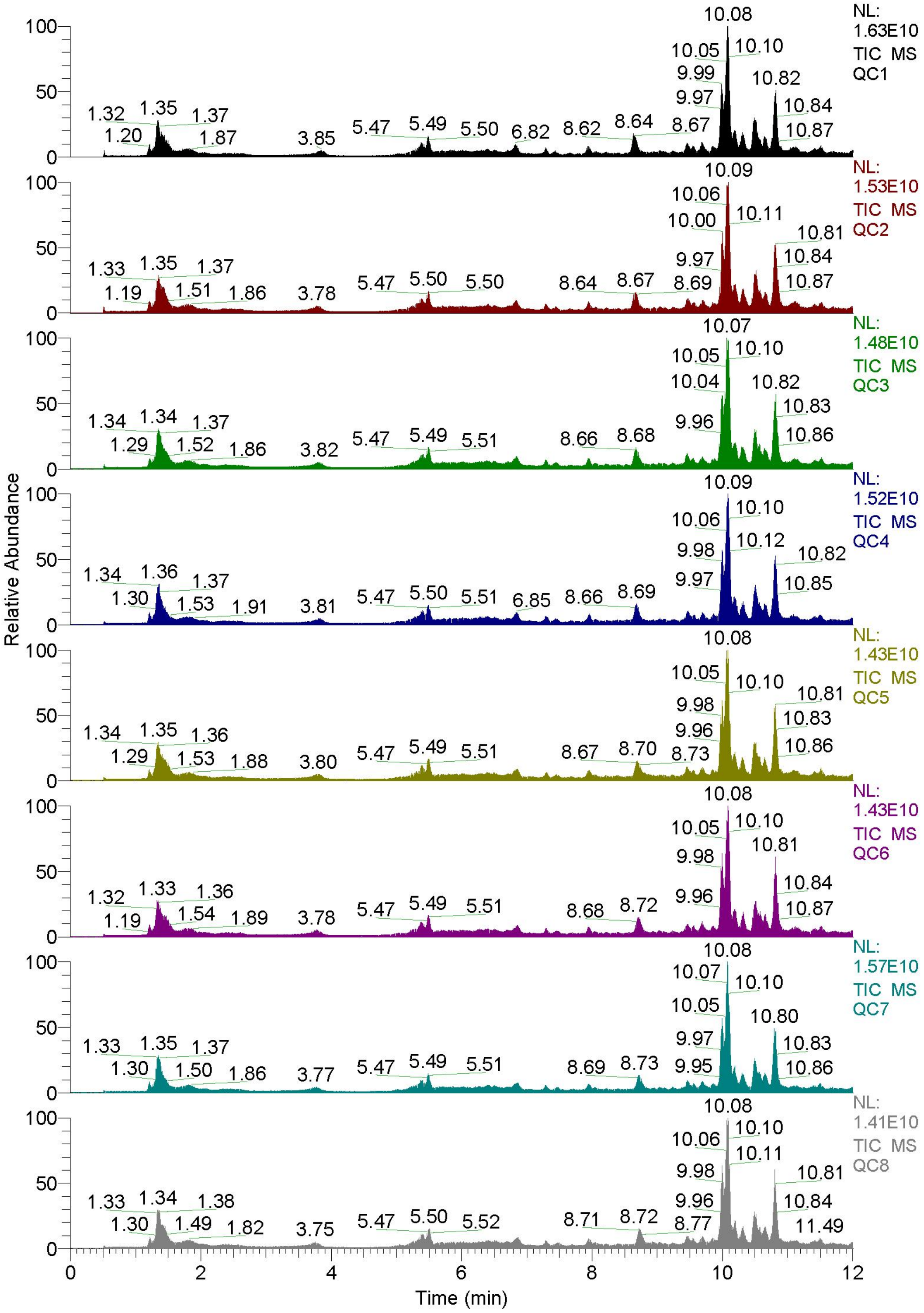

Supplementary Figure 6 The total ion chromatograms (TIC) of each uterine tissue sample captured in negative ion mode.
